# Vitamin K2 Prevents Lymphoma in *Drosophila*

**DOI:** 10.1038/s41598-017-17270-9

**Published:** 2017-12-06

**Authors:** Maytham A. Dragh, Zhiliang Xu, Zainab S. Al-Allak, Ling Hong

**Affiliations:** 1 Department of Genetics and Developmental Biology, College of Life Science and Technology, Huazhong University of Science and Technology, Wuhan, Hubei 430074 P. R. China; 2 Department of Biology College of Life Science, Misan University, Amarah, Iraq

## Abstract

Previous studies have established the anticancer effect of vitamin K2 (VK2). However, its effect on lymphoma induced by *UBIAD1/heix* mutation in *Drosophila* remains unknown. Therefore, we aimed to develop an *in vivo* model of lymphoma for the precise characterization of lymphoma phenotypes. We also aimed to improve the understanding of the mechanisms that underlie the preventative effects of VK2 on lymphoma. Our results demonstrated that VK2 prevents lymphoma by acting as an electron carrier and by correcting the function and structure of mitochondria by inhibiting mitochondrial reactive oxygen species production mtROS. Our work identifies mitochondria as a key player in cancer therapy strategies.

## Introduction

Vitamin K2 (VK2) is a fat-soluble vitamin that is important for human health. It is abundantly present in a variety of foods and usually exists in three forms: phylloquinone (VK1), menaquinone (VK2), and menadione (VK3)^1^.VK2 is produced by a vast array of bacteria^2^ and can be produced by animals and humans via the conversion of its other forms^3^.

The antitumor action of vitamin K has been investigated since 1947^4^. In rats, VK3 acts against adriamycin-resistant leukemia cells^5^. VK3, a radiosensitizing agent, extends the survival time of patients with bronchial carcinoma, and VK2 induces growth inhibition via cell cycle arrest^6,7^. VK2 exhibits remarkable antiproliferative effects on different cancer types^2,8^, including leukemia, lung cancer, ovarian cancer, prostate cancer, and hepatocellular cancer^9^. VK2 has anticancer effects against human bladder carcinoma^10^.

The protein product of *UBIAD1/heix* has multiple enzymatic activities, which include VK2 synthesis^3^, and menaquinone-4 synthesis in human^11^. *Drosophila heixudian* “*heix*” gene encodes a protein that bears high sequence identity with the human *UBIAD1* protein, loss-of-function of *UBIAD1* tends to progress bladder and prostate carcinoma^12^. Loss-of-function of *heix* leads to hemocyte overproliferation and aberrant differentiation^13^.*heix* mutants showed severe mitochondrial defects, and VK2 transfers electrons in *Drosophila* mitochondria, thus improving ATP production^14^.

During the larval stages of *Drosophila*, hemocytes are produced from a separate organ called the lymph gland^15^ and differentiate into two classes of cells: plasmatocytes and crystal cells^16^. Crystal cells are involved in the melanization of pathogenic material in the hemolymph^15^. They are clearly visible because of the Black cell (Bc) mutation, which causes premature melanization^17^. Moreover, crystal cells express the enzyme phenoloxidase (Pro-phenoloxidase A1, PO), which is responsible for the initiation of the melanogenesis cascade^18^. Melanin biosynthesis is induced by PO, which catalyzes the oxidation of phenols to quinones; quinones are subsequently polymerized into melanin^19^. Black spots, which are regarded as melanotic tumors or pseudotumors, are usually associated with crystal cells and are found in various *Drosophila* mutants^20^.

Previous studies have shown that hemocyte-mediated immune response is involved in larval melanization^21^. These immune responses include cell aggregation, phagocytosis, encapsulation, and melanization cascade induction^18^. Plasmatocytes and lamellocytes, which function in encapsulation, are rare in healthy larvae^22^. The activation of the JAK/STAT and immune-related pathways Toll and IMD is associated with the loss-of-function of *UBIAD1*/*heix* ^13^ in lymphoma and leukemia^23^.

Interestingly, significant similarities are found between the molecular mechanisms that regulate the development of the *Drosophila* lymph gland and the formation of the mammalian aorta–gonad–mesonephrous region^24^. The average levels of nucleotide diversity are tenfold lower in humans than that in *D. melanogaster* ^25^. A study that utilized next-generation sequencing to compare interspecies and intraspecies variation in humans and *Drosophila* revealed that 14.9% of human genes and 46.0% of fly genes have orthologs with a maximum identity of 45%^26^. A systematic analysis of human disease-associated gene sequences in *Drosophila* revealed that 79 out of 714 genes are associated with malignancies; among these genes, 29 are related with hematologic malignancies, including lymphomas, in humans^27^.

Lymphomas are tumors of hematopoietic and lymphoid tissues^28^ and can be classified as Hodgkin lymphoma or non-Hodgkin lymphoma (NHL)^29^. Approximately 90% of lymphomas are NHL^30^. NHL is one of the most commonly reported cancers in the United States and accounts for approximately 4% of all cancer cases. New cases of lymphoma account for approximately 72% of all cancer types, and approximately 3.4% of deaths in the United States can be attributed to blood cancers^31^.

This study is the first to apply VK2 in the treatment of *UBIAD1/heix* mutation-induced *Drosophila* Lymphoma LiD. We also aimed identify the mechanism that underlies the anticancer effects of VK2. Our study employed *Drosophila* as an *in vivo* model for the study of lymphomas, whereas related studies have utilized cancer cell lines. Thus, we aimed to characterize the lymphoma phenotype of *Drosophila* LiD.

## Results

### *UBIAD1/heix* mutation leading to development of lymphoma is prevented by VK2

**Figure 1:**
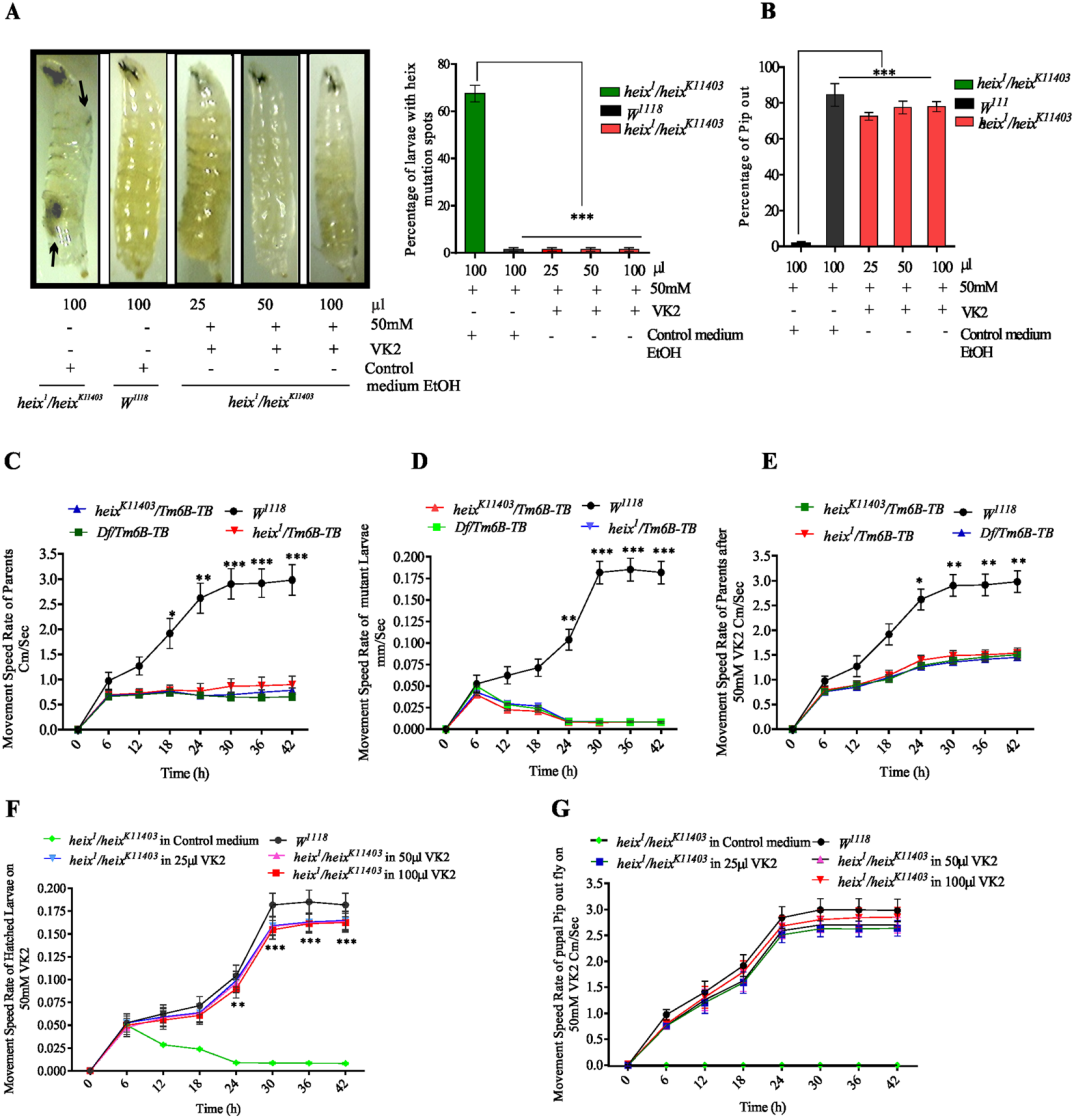
VK2 Prevents lymphoma in *Drosophila*. (**A**) Significant disappearance of black spots strongly evidenced with 25–100 µl of 50 mM VK2 dose. (**B**) Improvement of Pupal pip out rate. (**C**) Movement speed of *heix* mutant parent flies before treatment. (**D**) Movement speed of *heix* mutant larvae before treatment (**E**) Improvement in movement speed of mutant parent flies after treatment. (**F**) Movement speed Improved in larvae born from treated parent flies. (**G**) Movement speed of piped out flies fed on vitamin K2. Control media contain 50 mM Ethanol (EtOH) and treatment media contain 50 mM VK2. Means normalized to control (*Canton S*). Error bars indicate SEM. Analysis of variance (ANOVA/Dunnett: *P < 0.05, **P < 0.01, ***P < 0.001).

This is the first study to examine the therapeutic effects of VK2 on LiD induced by the *heix* mutation. Multiple doses of VK2 ranging from 1 mM to 50 mM were tested, and responses were associated with the disappearance of black spots (Fig. S2A) and improvement of the pupal pip-out rate (Fig. S2B). Dose volume was determined by selecting the volume that achieved the optimum vitamin distribution with the complete disappearance of black spots (Fig. S2C) and improvement of the pupal pip-out rate (Fig. S2D). Moreover, treatment with 30–50 mM VK2 improved movement speed (Fig. S2E). The complete and significant disappearance of black spots was achieved with 25–100 µl of 50 mM VK2 (***P < 0.001) (Fig. 1A). *heix* mutant larvae often die at the pupal stage^13^. Interestingly, in our research, third-instar larvae fed with VK2 showed significantly increased pip-out rates (***P < 0.001) and a strong response to VK2 (Fig. 1B). *heix* mutation is associated with impaired flight^14^ and reduced activity^13^. Similarly, we observed a significant decrease in movement speed (***P < 0.001) (Fig. 1C,D), which was restored by VK2 treatment (Fig. 1E) (See video in supplementary). We suggest that VK2 treatment increased energy supply, thus improving movement speed. Moreover, the rate of recently hatched larvae of parents fed and reared on VK2 significantly increased (***P < 0.001) (Fig. 1F). In addition, pupal pipped-out flies fed on VK2 showed significantly improved movement speed (Fig. 1G).

### VK2 inhibit ROS release

**Figure 2:**
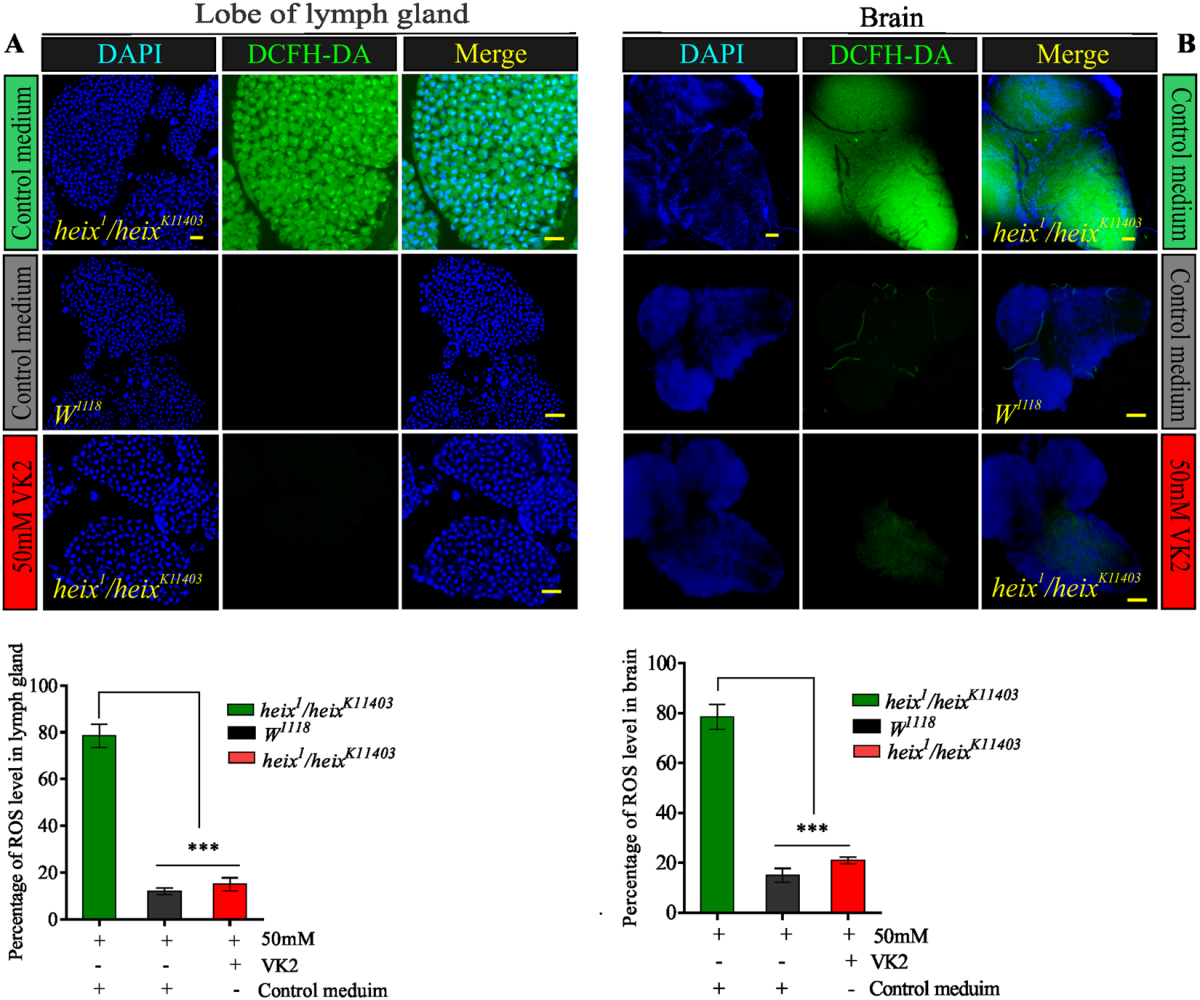
Vitamin K2 lowers ROS release. (**A**) Confocal microscopy showed increased ROS in Lymph gland of *heix* mutants, reduced by VK2 treatment. (**B**) Confocal microscopy showed increased ROS in Brain of *heix* mutants, reduced by VK2 treatment. Both lesions responded significantly to treatment. DAPI stain (blue) and ROS staining (CM-H2DCFDA) (green). Control media contain 50 mM Ethanol (EtOH) and treatment media contain 50 mM VK2. Means normalized to control (*Canton S*). Error bars indicate SEM. Analysis of variance (AVOVA/Dunnett: *P < 0.05, **P < 0.01, ***P < 0.001).

In accordance with the hypoxia, the phenotype of lymphoma specifically the loss of survival signs, is identical to the classical phenotype of hypoxic cancer cells^11^. The production of reactive oxygen species (ROS) is an index of cellular hypoxia^32^. We assessed ROS production in the lymph gland, a main target in the hematopoietic system. We also assessed ROS production in the brain given that brain cells are highly vulnerable to the deleterious effects, such as hypoxia, of ROS production during oxidative stress^33^. The results showed that the *heix* mutation is closely related to the loss of survival signs as a result of increased ROS production and decreased energy supply. ROS levels in the lymph gland (Fig. 2A) (***P < 0.001) and the brain (Fig. 2B) significantly decreased upon VK2 treatment (***P < 0.001). Mitochondrial defects associated with the *heix* mutation could be determined through mitochondrial ROS (mtROS) assessment. The functional status of the mitochondria could be inferred through the evaluation of ATP production.

### VK2 restores mitochondrial function by lowering mtROS and increasing ATP production

**Figure 3:**
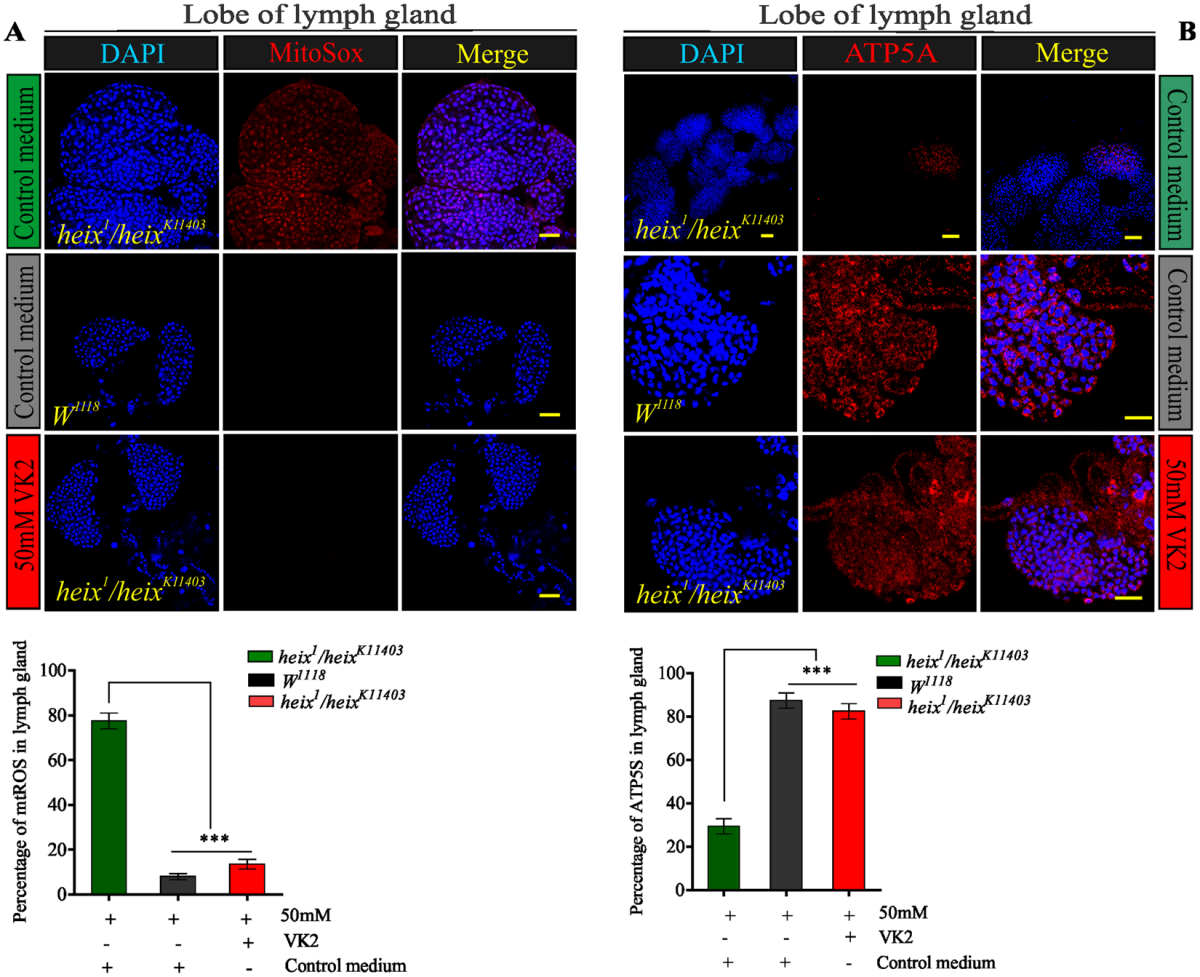
Vitamin K2 lowers mtROS and increases ATP production. (**A**) Confocal microscopy showed increased mtROS in Lymph gland of *heix* mutants, significantly reduced by VK2 treatment. DAPI stain (blue) and mitochondrial ROS staining (Mito-SOX) (red). (**B**) Confocal microscopy showed inhibition in ATP production in Lymph gland of *heix* mutants, significantly increased by VK2 treatment. DAPI stain (blue) and anti-ATP antibodies (ATP5A). Control media contain 50 mM Ethanol (EtOH) and treatment media contain 50 mM VK2. Means normalized to control (*Canton S*). Error bars indicate SEM. Analysis of variance (AVOVA/Dunnett: *P < 0.05, **P < 0.01, ***P < 0.001).

Mito-SOX was used to examine mtROS production. Our results revealed that mtROS amount increased in *heix* mutants, which responded positively and significantly to VK2 treatment (Fig. 3A). Furthermore, ATP production significantly (***P < 0.001) increased in the lymph glands of VK2-treated larvae (Fig. 3B). A similar response was observed in the brain (Fig. S2A and B). *heix* mutations are the main stimulants of MAPK (JNK and ERK1/2) pathways^13,34^; an evaluation confirmed that these pathways are activated in *heix* mutants. We then focused on the ability of VK2 to restore the normal activation of these pathways.

### VK2 is negative regulator of Ras/MAPK (JNK, ERK) and Toll, JAK/STAT, IMD pathways

**Figure 4:**
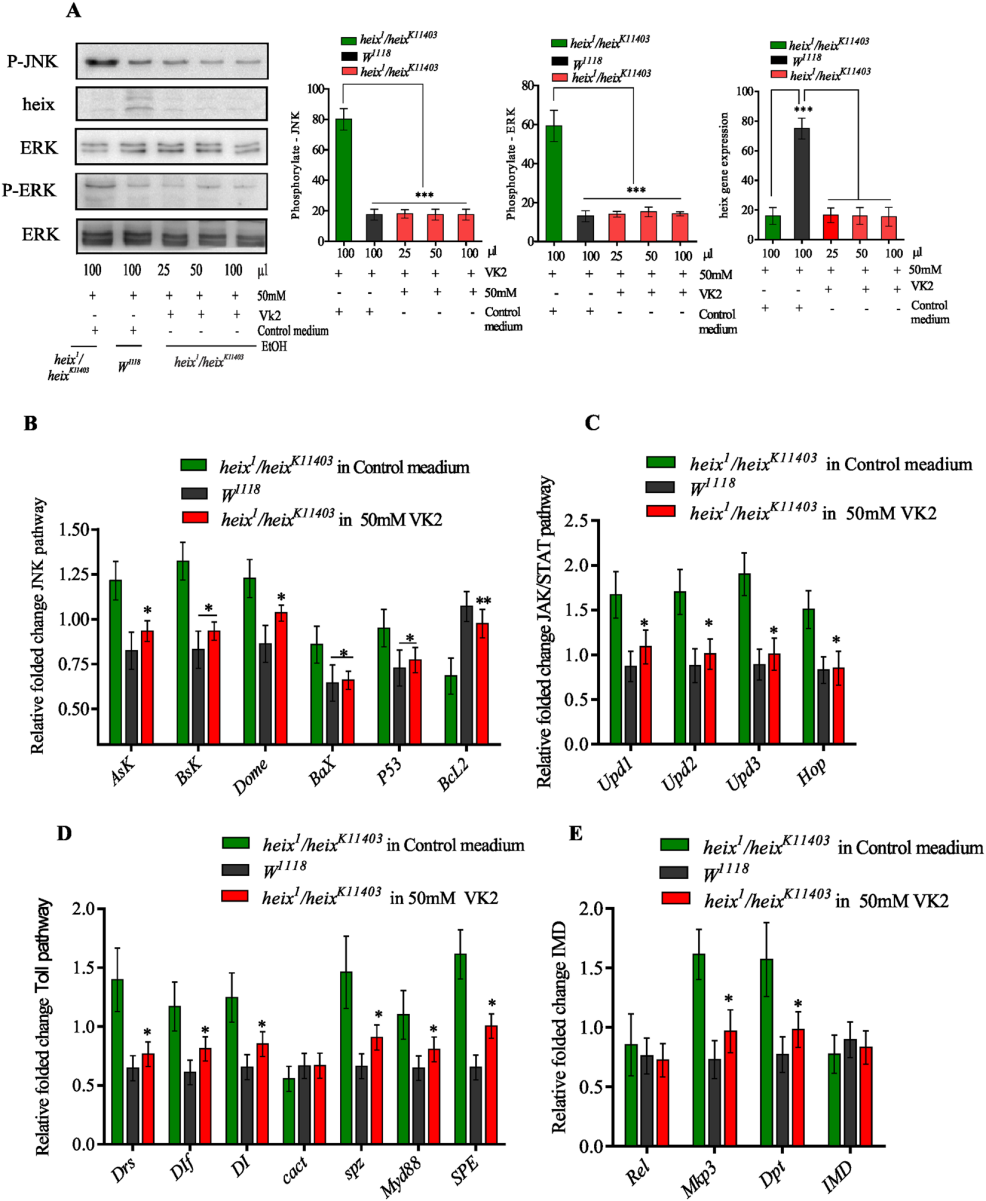
VK2 is negative regulator of Ras/MAPK (JNK, ERK) and Toll, JAK/STAT, IMD pathways. (**A**) Western blot analysis showed activation of JNK and ERK signaling pathways upon loss of function of *heix* gene and inhibition of activation significantly revealed after vitamin K2 treatment. (**B**) RT-PCR analysis of JNK pathway. (**C**) RT-PCR analysis of JAK/STAT pathway (**D**) RT-PCR analysis of Toll pathway. (**E**) RT-PCR analysis of IMD and Ras/MAPK pathway. Control media contain 50 mM Ethanol (EtOH) and treatment media contain 50 mM VK2. Transcripts were normalized to the housekeeping gene *Ef1α100E*. Error bars indicate SEM. A significant difference is compared to the control (AVOVA/Dunnett: *P < 0.05, **P < 0.01, ***P < 0.001).

We tested the activation of JNK and ERK to assess whether VK2 acts similarly as *heix* to negatively regulate MAPK pathways. VK2 treatment significantly inhibited activation (***P < 0.001) (Fig. 4A). Furthermore, we performed RT-PCR analysis to quantify the expression of several genes related to cell proliferation and cell death regulation. VK2 treatment modified the transcript levels of genes involved in the JNK pathway and related to mitochondrial function. Accordingly, the expression levels of genes, i.e., *Apoptotic single-regulating Kinase 1* (*P < 0.001), *basket* (*P < 0.001), *domeless* (*P < 0.001), *BCL2-associated X protein* (*P < 0.01), and *P53* (*P < 0.05), significantly increased, whereas the expression level of *B-cell CLL/lymphoma 2* (*Bcl2*) (**P < 0.001) strongly decreased; the expression levels of these genes were reversed following VK2 treatment (Fig. 4B).

Moreover, we performed RT-PCR analysis to quantify the expression levels of genes associated with the Toll, JAK/STAT, IMD, and Ras/MAPK pathways. These pathways have been proposed to regulate hemocyte proliferation and immune response^13,34^. VK2 treatment significantly decreased the transcripts of genes involved in the JAK/STAT pathway (*unpaired 1, 2, 3* (*Upds*) and *hopscotch* (*Hop*)) significantly decreased (*P < 0.05), (Fig. 4C). Furthermore, the expression of genes involved in the Toll pathway (*Drosomycin*, *Dorsal-related* immune factor, *dorsal*, *cactus*, *spatzle*, *myd88*, and *Spatzle-Processing Enzyme*) (*P < 0.05) (Fig. 4D). The expression of the genes of the IMD pathway (*diptericin A, B* (*Dpt*), *Relish* (*Rel*), and *immune deficiency* (*IMD*)) significantly decreased (*P < 0.01). In addition, the expression of the *mitogen-activated protein kinase phosphatase 3* (*Mkp3*), which is a gene involved with the Ras/MAPK pathway, significantly decreased (*P < 0.01) after VK2 feeding (Fig. 4E). *Dpt*, *Upds*, and *Mkp3* are often used as markers for the activation of the IMD, JAK/STAT, and Ras/MAPK pathways, respectively, and were highly responsive to VK2 treatment. As mentioned above, mitochondrial defects occur in *heix* mutation, and multiple transcripts are closely correlated with mitochondria. Mitochondrial function is closely associated with mitochondrial structure^35^.

### VK2 restores mitochondrial structure and membrane potential

**Figure 5:**
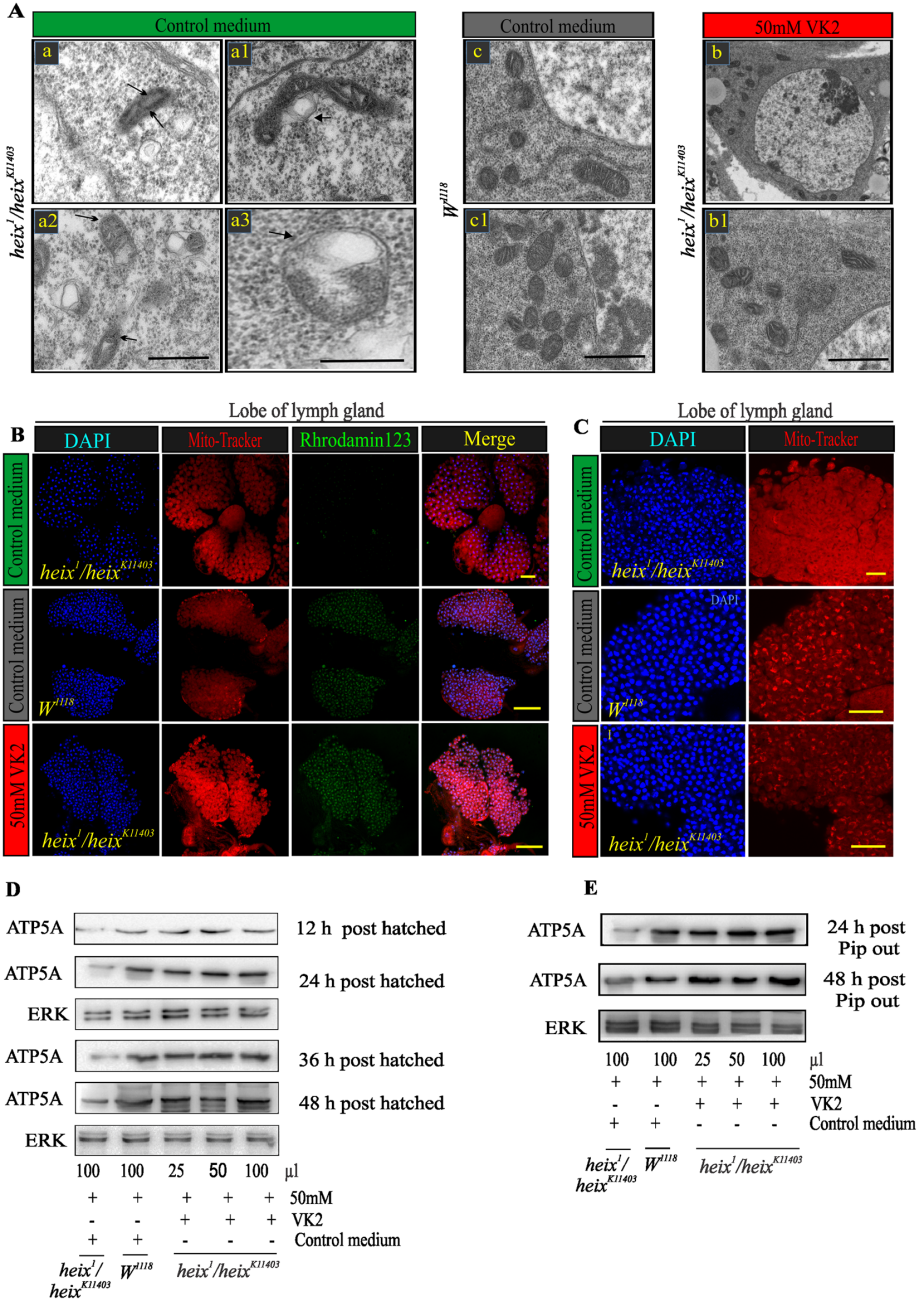
Vitamin K2 restores mitochondrial structure and membrane potential. (**A**) Transmission electron microscopy (TEM) images of lymph gland cells, *heix* mutant exhibits enlarged mitochondria with broken cristae, there are multiple swelling mitochondria of different size and several degrees of cristae disarrangement (arrow), higher magnification of a mitochondrion with electron-lucent matrix and subtotal Cristo lysis beside presence of amorphous metrical densities (arrows). Note the dilated mitochondrial matrix occupied by lipoid-like material (arrows). Method of staining: Uranile acetate/Lead citrate. Bar: 2 µm. (**B**) More negative mitochondrial membrane potential ΔΨm with VK2 treatment. DAPI stain (blue) and Rhodamin 123 (green). (**C**) Confocal microscopy of lymph gland labeled with Mito-Tracker showed mitochondrial morphological defects. DAPI stain (blue) and Mito-Tracker CMXROS (red). (**D**) Western blot analysis of recently hatched larvae (12, 24, 36 and 48 hrs post hatching) reared on vitamin K2 media, showed gradual increase in expression of ATP production. (**E**) Western blot analysis of pupal pip out flies (24 and 48 hrs post pupal pip out), gradual increase of ATP expression indicating response to VK2 treatment. Both (**D**) and (**E**) express response with 25–100 µl of 50 mM VK2. Control media contain 50 mM Ethanol (EtOH) and treatment media contain 50 mM VK2. Means normalized to control (*Canton S*). Error bars indicate SEM. Analysis of variance (ANOVA/Dunnett: *P < 0.05, **P < 0.01, ***P < 0.001).

Transmission electron microscopy (TEM) was performed to identify the relationship between mitochondrial function and structure and to verify the curative effects of vitamin K2 as an electron carrier. Mitochondrial swelling (MS) associated with the disarrangement of cristae and partial or total cristolysis are the most consistent submicroscopic alterations observed in this study. The majority of mitochondria were round in shape with an electron-lucent matrix, subtotal cristolysis, and the presence of amorphous metric densities with a dilated mitochondrial matrix occupied by lipid-like material (Fig. 5A). VK2 treatment restored mitochondrial structure, thus restoring normal mitochondrial function.

Additionally, negative mitochondrial membrane potential (ΔΨm) significantly increased following VK2 treatment (Figs 5B, S4 and S5). Interestingly, Mito-Tracker labeling revealed that mitochondrial morphological defects and dislocation significantly increased in *heix* mutant which responded to VK2 treatment (Figs 5C, S4 and S5).

We assessed ATP production in parent flies before and after VK2 treatment and found interesting differences (Fig. S5C). The results indicated that VK2 efficiently transferred electrons in the mitochondrial membrane, thus promoting efficient ATP production. The effective VK2 dose that increased ATP production was also determined and ranged from 30 mM to 50 mM (Fig. S5D). ATP production at 12, 24, 36, and 48 h after egg hatching under VK2 treatment gradually increased (Fig. 5D). This improvement was significant (*P < 0.01) after 12 and 24 h post-hatching and highly significant (***P < 0.001) at 36 and 48 h post-hatching (Fig. S4). ATP production increased at 24 and 48 h after the pupal pip out of flies both measurements were significant (***P < 0.001) (Figs 5E and S4).

### VK2 prevents lymphoma phenotype caused by *heix* mutation

**Figure 6:**
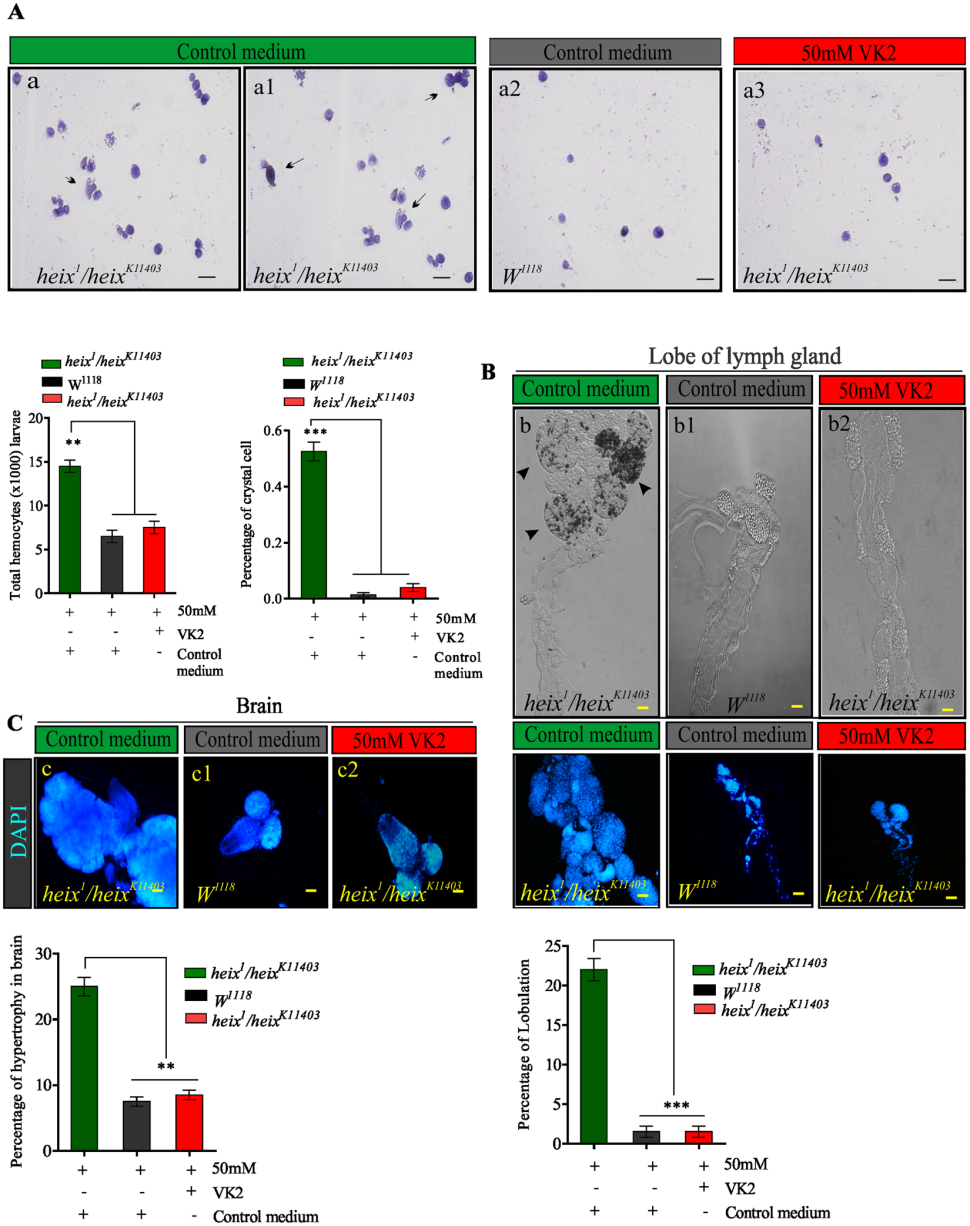
Vitamin K2 rescues hematopoietic system in *Drosophila* third instar larvae with lymphoma induced by *heix* mutation. (**A**) Images (X40) circulating hemocytes labeled by Giemsa staining kit from larvaes in *heix* ^*k11403*^ */heix* ^*1*^ on control medium, in control (*Canton S*) on control medium, and in *heix* ^*k11403*^ */heix* ^*1*^ on VK2 supplemented medium. Images reveal an increase number of crystal cells, (black arrows). Total circulating hemocytes counted from at least twenty third instar larvaes of each genotype. The proportion of crystal cell observed in total circulating hemocytes. (**B**) Images (X20) lymph glands in third instar larvaes in *heix* ^*k11403*^ */heix* ^*1*^ on control medium, in control (*Canton S*) on control medium and in *heix* ^*k11403*^ */heix* ^*1*^ on VK2 supplemented medium. They show a significant increase size of mutant lymph glands indicating hyperplasia which responded to treatment with VK2. Percentage of lobulation in lymph gland reduced significantly. (**C**) Images (X20) brain in third instar larvae in *heix* ^*k11403*^ */heix* ^*1*^ on control medium, in control (*Canton S*) on control medium and in *heix* ^*k11403*^ */heix* ^*1*^ on VK2 supplemented medium. They show a significant hypertrophy in mutant brains which responded to treatment with VK2. Control media contain 50 mM Ethanol (EtOH) and treatment media contain 50 mM VK2. Means normalized to control (*Canton S*). Error bars indicate SEM. Analysis of variance (ANOVA/Dunnett: *P < 0.05, **P < 0.01, ***P < 0.001).

Black spots, which indicate increased hemocyte proliferation, completely disappeared after VK2 treatment. Therefore, we prepared hemocytic smears using *Giemsa* staining to show the rescue of hemocyte proliferation following VK2 treatment. The percentages of total hemocytes (**P < 0.01) and crystal cell counts significantly decreased (Fig. 6A). The results also revealed hyperplasic lymph glands with numerous lobes. VK2 treatment, however, restored the normal structures of lymph glands, and the percentage of lobulation significantly decreased (***P < 0.001) (Fig. 6B). Furthermore, brain hypertrophy also responded positively to VK2 treatment (Fig. 6C).

### Prevention of lymphoma is VK2 dependent

**Figure 7:**
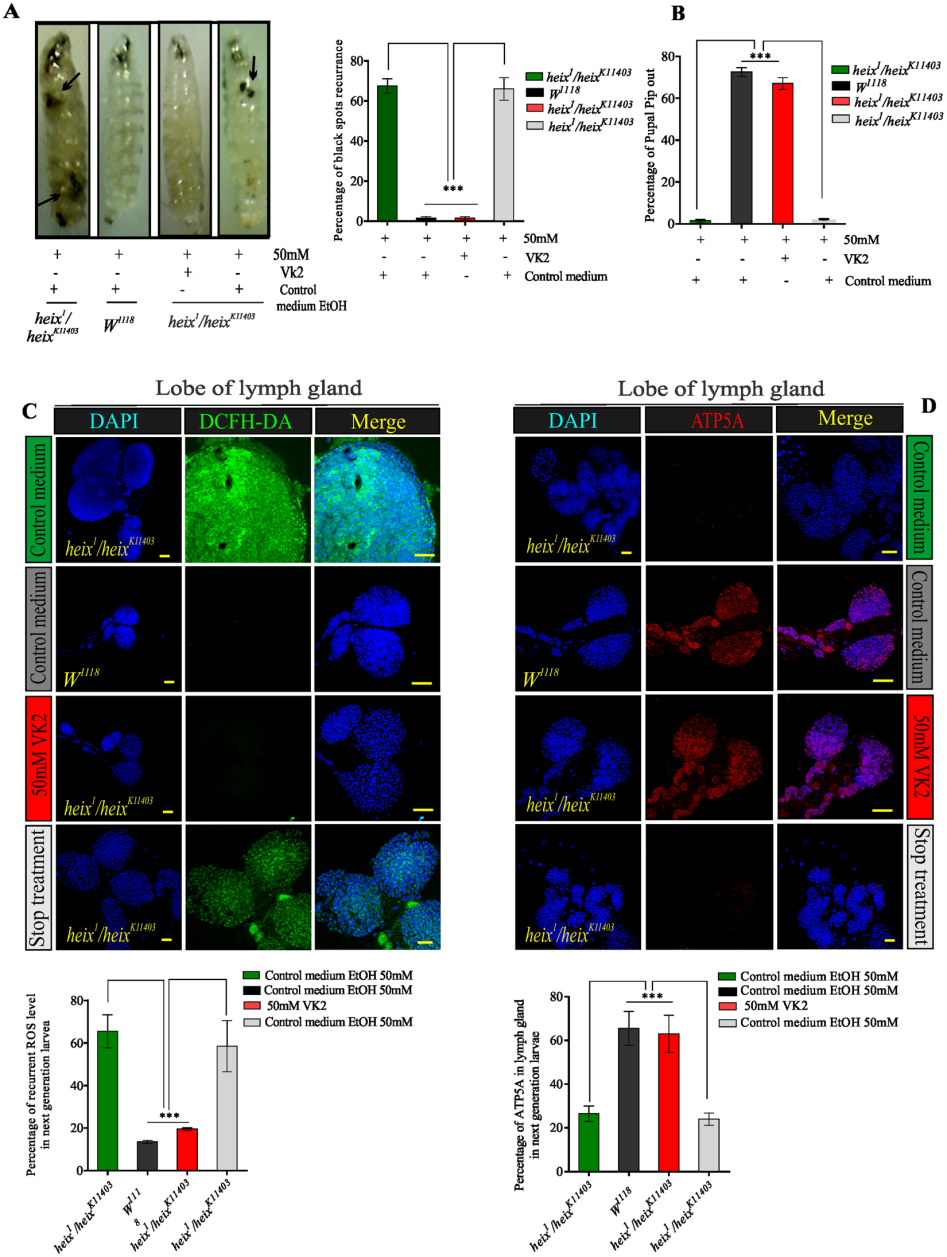
Prevention of lymphoma was vitamin K2 dependent. Testing the ability of VK2 to reduce recurrence of lymphoma. Follow-up of second-generation flies fed on VK2, divided into two groups: one group continuously reared on VK2 medium and the other on ordinary control medium. (**A**) Reappearance of black spots after stopping VK2 treatment. The percentage of larvae with recurrent spots very high. (**B**) Pupal pip out rate of adult flies decreased after stopping VK2 treatment. (**C**) ROS expression clearly increased after stopping VK2 treatment, recurrence of ROS release was significant. DAPI stain (blue) and ROS staining (CM-H2DCFDA) (green). (**D**) Inhibition of ATP production after stopping VK2 treatment, decrease in ATP production was significant. DAPI stain (blue) and anti-ATP antibodies (ATP5A). Control media contain 50 mM Ethanol (EtOH) and treatment media contain 50 mM VK2. Means normalized to control (*Canton S*). Error bars indicate SEM. Analysis of variance (ANOVA/Dunnett: *P < 0.05, **P < 0.01, ***P < 0.001).

We followed up second-generation larvae born from flies fed with VK2. The larvae were divided into two groups. One group was reared on VK2 medium and the other was reared on ordinary control medium (more details are shown in Fig. S6). Surprisingly, the lymphoma phenotype recurred in the second group, which exhibited the reappearance of black spots with significantly high percentage of larvae with recurrent spots (***P < 0.001) (Fig. 7A). Larvae in the first group had a higher pupal pip-out rate than those in the second group (***P < 0.001) (Fig. 7B). Furthermore, ROS levels significantly increased (***P < 0.001) in the lymph glands of larvae in second group but were lower in larvae reared on VK2 medium (Fig. 7C). Similar results were obtained for the brain (Fig. S7A). ATP production was inhibited in larvae in the second group (Fig. 7D). The same results were demonstrated in the brain (Fig. S7B). This experiment proved that VK2 can prevent the progression, but not recurrence, of lymphoma.

### Prevention of lymphoma is VK2 related

**Figure 8:**
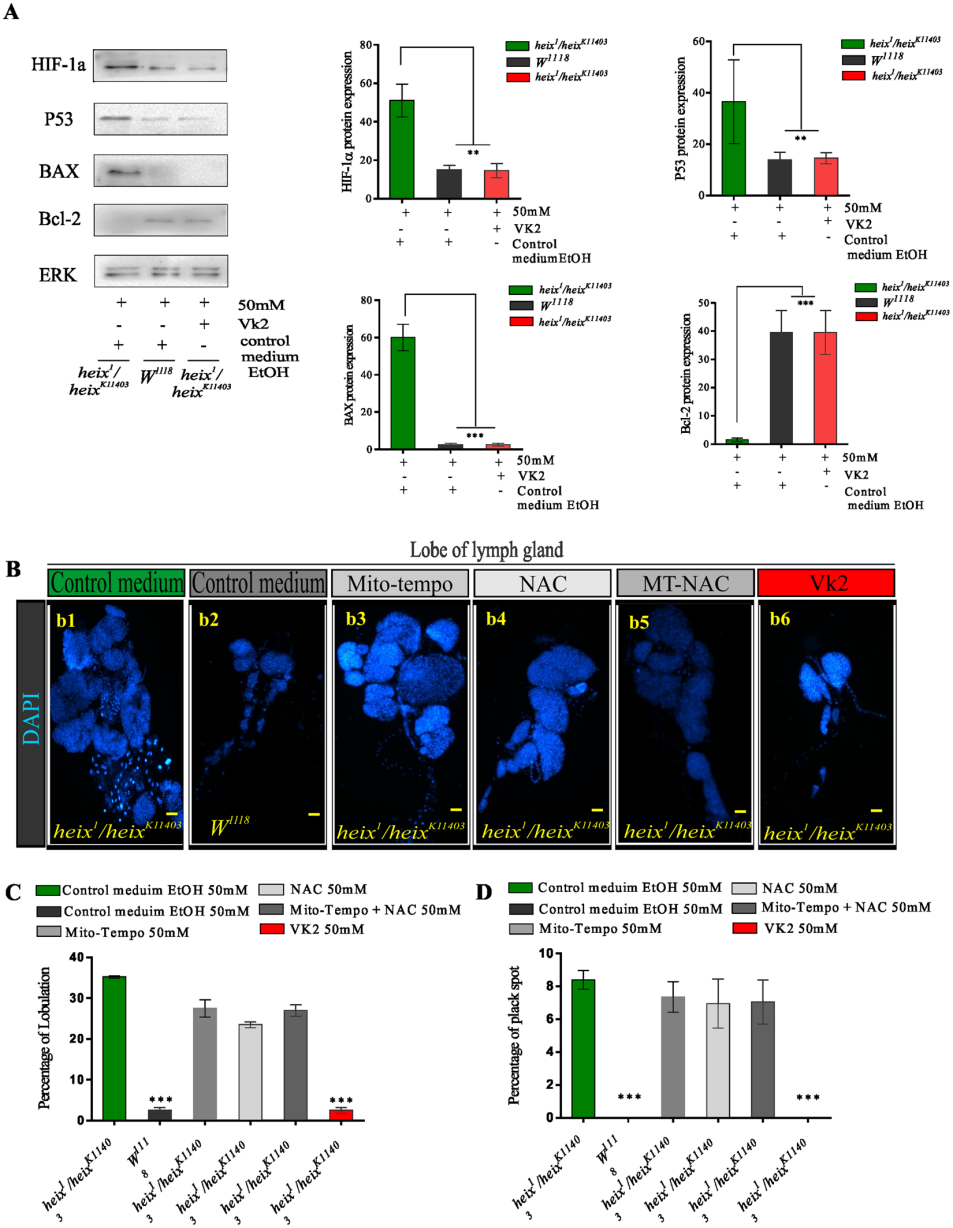
Responses revealed were due to VK2 treatment. Loss of function of *heix* lead to mitochondrial defects represented by hypoxia. (**A**) Assessment of *HIF* protein expression by western blot analysis for conformation of hypoxia condition, distinct expression of *HIF* protein in *heix* mutants (*heix* ^*k11403*^ */heix* ^*1*^) reared on control medium compared with a significant lesser expression in both control (*Canton S*) and VK2 treated groups. The difference in expression level quite significant. (**A**) Expression of mitochondrial regulator genes (*Bcl2* family), a western blot including *Bcl2, Bax*, and *P53*, there is clear expression of *Bax* and *P53* and fair expression of *Bcl2* in *heix* mutants, all responded to treatment with VK2, and reversed expression was revealed. Response was significant with *P53* protein, more significant with both *Bax* and *Bcl2*. (**B**) Treatment of *heix* mutant larvae with antioxidants N-Acetyl-L-cysteine (NAC) and (Mito-TEMPO) (MT) scavengers in three treatments group design, one with NAC, the other with MT and the third with both of them. *heix* mutant on control medium with distinct hyperplasia of lymph gland, control (*Canton S*) on control medium with normal lymph gland, *heix* mutant treated with MT scavenger showed lymph gland hyperplasia, *heix* mutant treated with NAC scavenger also showed lymph gland hyperplasia, *heix* mutant treated with MT + NAC scavengers revealed lymph gland hyperplasia, *heix* mutant treated with VK2 showed normal lymph gland. (**C**) Scavengers didn’t reduce the high lobulation of *heix* mutant lymph gland as VK2 achieved. (**D**) The percentage of black spots in *heix* mutant larvae didn’t affected by scavenger’s treatment as demonstrated with VK2 treatment which showed complete disappearance of them. Control media of control (*Canton S*) and *heix* mutant contain 50 mM Ethanol (EtOH), and treatment media contain 50 mM of each scavenger’s. Error bars SEM. A significant difference is compared to the control (*Canton S*) (ANOVA/Dunnett: *P < 0.05; **P < 0.01; ***P < 0.001).

We assessed *HIF* protein expression through western blot analysis to confirm the occurrence of hypoxia. *heix* mutants reared on control medium exhibited distinctive *HIF* protein expression, whereas control and VK2-treated groups had significantly lower (**P < 0.001) *HIF* expression (Fig. 8A). Hypoxia and elevated cytosolic ROS and mtROS indicate serious mitochondrial defects; thus, investigating the protein expression of mitochondrial regulator genes (*Bcl2* family)^36^, provide evidence for the mitochondrial dysfunction hypothesis we suggested in our study and for the role of VK2 in the rescue of lymphoma phenotypes. Western blot analysis was performed on *Bcl2*, *Bax*, and *P53*. *heix* mutants exhibited the clear expression of *Bax* and *P53* and fair expression of *Bcl2*. VK2 treatment reversed the expression of these proteins, the response of *P53* was significant (**P < 0.001) and those of *Bax* and *Bcl2* were highly significant (***P < 0.001) (Fig. 8A).

Previous studies used specific cytosolic ROS scavengers (*N-acetyl-L-cysteine*) (NAC) to evaluate the specificity of the effects of their tested treatment^10^; other studies used mtROS scavengers (*2(2,2,6,6-tetramethylpiperidin-1-oxyl-4-ylamino)-2-oxoethyl*) *triphenylphosphonium chloride* (Mito-TEMPO) to evaluate the tumor-suppressing effects of antioxidant compounds^37^. To demonstrate that all responses, especially inhibition of cytosolic and mtROS production, are specific to VK2 treatment, we treated *heix* mutant larvae with NAC and Mito-TEMPO scavengers. We then divided the larvae into three treatment groups; i.e., one treated with NAC, the other with Mito-TEMPO, and the third with NAC and Mito-TEMPO. Interestingly, larvae did not show high response to scavenger treatments. Scavenger-treated larvae showed attenuated lymph gland hyperplasia; this effect, however, was not significantly different from that shown by VK2-treated larvae (Fig. 8B). In contrast to VK2, scavengers did not decrease the high lobulation of *heix* mutant lymph glands (Fig. 8C). Moreover, black spots in *heix* mutant larvae, which were unaffected by VK2 treatment, completely disappeared (Fig. 8D). Blood smears from the three treatment groups showed the persistent proliferation of hemocytes and an increased number of crystal cells, whereas those from control and VK2-treated larvae showed normal blood characteristics (Fig. S8). Furthermore, the increased number of total hemocytes (Fig. S8B) and percentage of crystal cell (Fig. S8C) of the scavenger-treated larvae were significantly (***P < 0.001) different from that of control and VK2-treated larvae. This result indicated that VK2 treatment regulates the hematopoietic system and restores normal blood cell count, whereas the scavenger treatment failed to regulate hemocyte proliferation.

## Discussion

This study was designed to highlight the important role of VK2 in lymphoma treatment, as well as to provide insight on the mechanisms that underlie the curative effect of VK2. Interestingly, our results strongly indicated that VK2 effectively prevents lymphoma progression.

We found that *heix* mutant *Drosophila* exhibited almost all classical lymphoma phenotypes, including lymph gland hyperplasia, brain hypertrophy, hemocyte overproliferation, melanotic black spots, and poor survival signs. VK2 treatment rescued black spot formation, which is the most obvious feature of lymphoma in *Drosophila*. Other studies have reported that the loss-of-function of *UBIAD1/heix* leads to the activation of immune-related pathways and hyperplasia of lymph glands^13^. Hemocyte proliferation is closely correlated with the formation of black spots, which can be attributed to an increase in number of crystal cells associated with melanization^17,38^. Hence, the disappearance of black spots upon VK2 treatment may be attributed to the regulation of hemocyte proliferation. The activated form of *Hop* interacts with the Ras/MAPK pathway and hemocyte proliferation^39^, which is positively triggered in *heix* mutant larvae^13,40^. This finding is consistent with the significant upregulation of *Hop* expression in the *heix* ^*k11403*^ mutant. Our results demonstrated the ability of VK2 to restore lymphoma-associated alterations and indicated that similar to the *heix* gene, VK2 can act as a negative regulator of immune-related and cell-proliferation-associated pathways.

Furthermore, recent studies have demonstrated that the *heix* mutation is associated with certain acetifications, such as impaired flight^14^ and limited physical and biological activity^13^. Biological activities require energy, and their alterations were rescued by VK2 treatment. This recovery resulted from the increase in energy supply, which is closely associated with the restoration of mitochondrial function. These findings indicated that VK2 is a key player in the restoration of mitochondrial function.

Our data also demonstrated that cytosolic ROS and mtROS production increased in *heix* mutant larvae. A recent study by Mugoni *et al*. showed that silencing *UBIAD1* increases ROS production^41^. Under normal conditions, mitochondria receive pyruvate from the cytosol, where oxidative phosphorylation occurs, to produce ATP through the tricarboxylic acid cycle and electron transport chain (ETC)^42^. Conversely, under hypoxia, cells utilize anaerobic glycolysis, which converts pyruvate into lactate and rapidly produces ATP^43^. Although most electrons that pass through the respiratory chain are transferred to oxygen at complex IV, some electrons leak from complexes, particularly complexes I and III, and then react with molecular oxygen to produce superoxide^44^. Superoxide is converted to hydrogen peroxide by Mn–superoxide dismutase^42^ and can freely diffuse across the mitochondrial membrane into the intermembrane space and cytosol^45^. Defects in the mitochondrial respiratory chain increase ROS production^34^. These modifications promote the kinase signaling cascade-like activation of MAPK (JNK and ERK)^45^. VK2 likely exerts its anticancer activity by restoring mitochondrial function through inhibiting ROS production.

Given that *heix* mutants suffer from serious defects in ETC^14^, the partial inhibition of ETC leads to excessive mtROS production^46^. mtROS are natural byproducts and are key steps in the generation of ATP^44^. Our results showed that VK2 decreased mtROS production and restored ATP production by acting as an electron carrier in the mitochondrial membrane.

The general role of ROS in cancer cells is a highly controversial subject. Intracellular signaling pathways, such as MAPK, are triggered by ROS^47^. Increased levels of ROS with hypoxia are direct factors that activate cell proliferation; signaling; and immune-related pathways, such as Toll, IMD, JAK/STAT, and JNK^47,48^. JNKs catalyze the phosphorylation and downregulation of *Bcl-2* proteins^48^. *Bcl-2* antagonizes ROS generation^49^. JNK alters the composition of the *Bax/Bcl-2* complex by increasing *Bax* expression, which leads to the formation of *Bax* homodimers and in the disruption of mitochondrial membrane integrity^50^. ERK1/2 and JNK are activated through *Ask-1*; *heix* mutants showed the clear activation of these pathways. Moreover, ERK is associated with cell proliferation^51^.

We observed that all *Bcl-2* family proteins, except for the *Bcl-2* transcript, were significantly overexpressed in *heix* mutants. *Bcl-2* proteins regulate ETC in the mitochondrial membrane^36^. The Ras/MAPK pathway is triggered in *heix* mutants, and *UBIAD1/heix* is a negative regulator of this pathway^40^. We also demonstrated the roles of related genes associated with MAPK pathways, such as *MKP3* and *Dpt*, with *Hop*.

The activation of the JAK/STAT signaling pathway is associated with numerous malignancies, including lymphoma and leukemia^23^. Xia *et al*. detected the remarkably high expression of *Upd3* in the JAK/STAT pathway of *heix* mutant larvae^13^. Our work showed high expression of *Upds* in the JAK/STAT pathway.

Our results indicated that *heix* mutations induce the aberrant activation of IMD and Toll pathways. The IMD pathway controls the expression of antimicrobial peptides^52^. We showed that activation of the IMD and Toll pathways is indicative of a hematopoietic disorder. VK2 feeding restored the normal expression of included transcripts.

The structure of the mitochondria is closely related to its function, and mitochondrial structure and functions vary from tissue to tissue^53^. Silencing *UBIAD1* causes dramatic morphological changes and cholesterol storage in the mitochondria; this effect thus emphasizes the important role of *UBIAD1* in mitochondrial function^54^. Interestingly, mutations in the *Drosophila heix* gene resulted in abnormal mitochondrial morphology and malfunction^13,14^. TEM sections revealed that MS is associated with the disarrangement of the cristae and with partial or total cristolysis. This morphological change is associated with hypoxic–ischemic conditions^55^.

Partial or total cristolysis is suggestive of the severely compromised ability of cells to generate ATP^56^. VK2 treatment, however, can restore normal mitochondrial structures by modifying the membrane potential of mitochondria while acting as an electron carrier. The application of this vitamin recharged the mitochondrial membrane and increased its negativity. ETC, which is located in the mitochondrial inner membrane (MIM), pumps protons out of the mitochondrial matrix into the intermembrane space between the MIM and mitochondrial outer membrane. This process slightly changes pH and increases membrane potential due to the charge separation across MIM and the absence of anions that accompany positively charged protons. Thus, ΔΨm across MIM is negative on the matrix side. Therefore, proton-motive force resulting from membrane potential and pH gradient regulates ATP production from ADP and phosphate by ATP synthase^57^.

We evaluated the inhibitory effect of VK2 on the progression and recurrence of lymphoma in *Drosophila*. When VK2 feeding was stopped, most lymphoma phenotypes recurred. We found that VK2 prevents lymphoma by acting as a mitochondrial electron carrier, which is necessary for establishing mitochondrial membrane potential and facilitating ATP production.

Finally, to determine that the restoration of mitochondrial function and structure were specific to VK2 treatment, we introduced ROS scavengers, which are antioxidants that discard free radicals^58^. ROS scavengers suppress tumor growth^37^; therefore, responses to these compounds indicate responses that are not specific to VK2. In this study, the nonsignificant response to ROS scavengers revealed that the restoration of mitochondrial function can be attributed toVK2 treatment.

Our work indicates that VK2 prevents the progression, but not recurrence, of lymphoma. These findings were in agreement with those of previous studies that were unable to confirm the efficacy of VK2 in suppressing HCC recurrence^59,60^. However, other studies have shown that VK2 can decrease cancer recurrence^61,62^. Therefore, further studies in this regard are still needed.

In conclusion, we confirmed that VK2 prevents the progression, but not recurrence, of lymphoma by restoring mitochondrial function. Our study suggests that the correction of mitochondrial function might be a fundamental step in tumor treatment and identifies VK2 as a key player in cancer therapy strategies.

## Materials and Methods

### Fly stocks and husbandry

All flies raised on yeast/molasses-based food at 25 °C on a 12-hr light/dark cycle, unless otherwise noted, according to standard procedures. *Canton S* (*W* ^1118^) (Bloomington stock 1) used as control, and two *heix* alleles used: first p-element allele (*heix* ^*K*1*1403*^) (Bloomington stock11031) and second ethyl methane sulfonate (EMS) allele (*heix* ^1^) (Bloomington stock 3600). To identify *heix* phenotype, deficiencies: *Df (2 L) RA5/Cyo* (Bloomington stock 6915) used to identify *heix* phenotype. *TheSp*, *CyO*, *Mkrs*, and *TM6 Band Sm6*/*Tm6B* balancers used for phenotype identification (kindly provided by Zhaohui Wang, Institute of Genetics and Developmental Biology, Chinese Academy of Sciences, China). Mutant flies were obtained by mating *heix* ^*k11403*^ and *heix* ^1^ or *Df (2 L) RA5/Cyo*.

### Media preparation of *Drosophila* melanogaster

Methyl-p-hydroxybenzoate (C_8_H_8_O_3_) 10 g was dissolved in 100 ml of ethanol and configured to be a 10% methyl-p-hydroxybenzoate acid solution. Weigh the corn flour 120 g, agar 5 g, brown sugar 40 g into the pot, add water to 1 L, heat on the induction cooker, stir slowly in the heating process until the boiling. Add 10 ml of 10% methyl-p-hydroxybenzoate acid solution to 1 L of food, and add 4.3 ml of propionic acid, both to prevent fruit fly food spoilage. Pour the food into a bottle; stored in RT, it is ready for use, the flies were bred in a constant temperature and humidity incubator, temperature 25 C°, humidity 60%.

### Vitamin K2 feeding

The newly generated genotype *P-element* allele, *heix* ^*k11403*^, an EMS allele, *heix* ^*1*^, and *Df* adult virgin flies (not more than 6 h old) were placed on medium supplemented with VK2 (Sigma USA) (VK2 was dissolved in 99.9% ethanol, EtOH). The therapeutic dose determined by testing several gradual concentrations of VK2, from 1 mM to 50 mM, and identifying optimum response based on black spots disappearance (Fig. S2). Determination of dose volume ranging from 1 µl to 35 µl, were tested. The 25-µl volume showed an even distribution of vitamin on medium (2 cm in diameter &1 cm height) (Fig. S2). Adult virgin flies put in medium with VK2 for 24 hrs. Flies were placed together for mating in new tubes with VK2 for 4–5 days then removed before the eggs hatched. Only long “*heix* mutant” larvae were selected (100 larvae per group) for rearing and growth on VK2. Both control “*Canton S*, *W* ^1118^ and *heix* mutant” controls were reared on medium followed by the addition of 50 mM ethanol (EtOH). Molasses medium incubated for 24 h uncovered at room temperature (RT) to allow evaporation of ethanol and, thus, avoid alcohol-induced stress^63^. (The experiment was repeated several times to validate the results).

### Immunostaining of *Drosophila* larval tissues

*Drosophila* third instar larvae were dissected in phosphate-buffered saline solution (1x PBS). Lymph glands and brains immediately transferred into a container of PBS and placed on ice, followed by fixation with 4% paraformaldehyde for 10–15 minutes at RT and three washes with PBST. Samples blocked in PBS +Triton–x100 0.3% +1% BSA for 1 h at RT, followed by staining with ab13847 at a 1/500 μl dilution (in PBS +Triton-x100 0.3% +1% BSA) for 16 h at 4 °C. The secondary antibody, Alexa Flour 594 WGA (reddish color), used to label plasma membrane at a 1/500 dilution for 1 hr. DAPI used to stain cell nuclei (blue) at a concentration of 1.43 µM. For ROS detection, 2′, 7′-dichlorofluorescein-diacetate (DCFH-DA) utilized. DCFH-DA, a cell membrane-permeable dye, is converted to DCFH (a non-fluorescent cell membrane-impermeable compound, Invitrogen Molecular Probes, cat no. C6827). Tissues incubated with dye for 10–15 minutes in a dark chamber on an orbital shaker at RT followed by three 5-minutes washes in 1x PBS on an orbital shaker at RT, fixation for 4–8 minutes in 7% formaldehyde in 1x PBS, and rinsing in 1x PBS immediately after fixation. Images captured using an Olympus confocal laser scanning microscope FV1000. Images were analyzed using ImageJ software and processed by Adobe Photoshop and Corel DRAW.

### Mitochondrial superoxide indicator

Tissues stained with 5 μM Mito-SOX™ prepared from 5 mM Mito-SOX™ reagent stock solution. The contents (50 μg) of one vial of Mito-SOX™ mitochondrial superoxide indicator (Component A) were dissolved in 13 μL of dimethyl sulfoxide (DMSO) to make the 5 mM Mito-SOX™ reagent stock solution. The 5 mM Mito-SOX™ reagent stock solution was dissolved in HBSS/Ca/Mg to prepare a 5 μM Mito-SOX™ reagent working solution. Then, 0.3–0.5 µl of the 5 μM Mito-SOX™ reagent working solution was used to cover the tissues. Tissues were incubated for 10 minutes at 37 °C in the dark. Tissues then washed gently three times with warm buffer. DAPI at a concentration of 1.43 µM used to stain cell nuclei (blue). Tissues fixed for 5 minutes in 4% formaldehyde and rinsed twice in 1x PBS immediately after fixation, then mounted for microscopy.

### Mitochondrial membrane potential assay

Changes in mitochondrial membrane potential in tissues evaluated using Rhodamine 123, a mitochondrial-specific dye. Briefly, sample stained with 1.5 μM Rhodamine 123 and incubated at 37 °C for 10 minutes. Sample subsequently washed three times with warm PBS to remove unbound dye, fixed for 5 minutes in 4% formaldehyde then rinsed twice in 1x PBS after fixation. DAPI applied at 1.43 µM to stain cell nuclei (blue). Mitochondrial membrane potential assay evaluated by fluorescence of Rhodamine 123 under an Olympus confocal laser scanning microscope FV1000.

### MitoTracker® Red CMXRos Mitochondrial Probe

MitoTracker® Stock Solution dissolved in high-quality anhydrous dimethyl sulfoxide (DMSO) to a final concentration of 1 mM. The 1 mM MitoTracker® stock solution diluted for live-cell staining, and concentrations of 100–500 nM used. Lymph glands transferred in to PBS; after removal of PBS from tissues, MitoTracker® added and incubated for 15–20 minutes at RT. Tissues washed three times with PBS, fixed for 5 minutes in 4% formaldehyde and rinsed twice in 1x PBS. DAPI stain (blue). MitoTracker® Red evaluated by fluorescence under an Olympus confocal laser scanning microscope FV1000.

### Western blot analysis

Total protein from third instar larvae extracted by homogenization with (PRO PREP) protein extraction kit (INTRON Biotechnology, Korea). Samples subjected to 1x SDS-PAGE loading buffer and separated by 12% SDS-PAGE. Then transferred to a polyvinylidene fluoride (PVDF) membrane for immunoblotting. Membranes blocked in 5% non-fat dry milk in TBST (0.05% Tween, 20–50 mmol/L Tris HCL, pH 7.5 and 150 mmol/L NaCl) for 3 h, and incubated overnight with primary antibody at 4 °C and washed with TBST for 2 h. Secondary goat anti-rabbit IgG (HRP) incubated with membranes at RT for 3 h. After incubation, samples washed with TBST for 2 h. Finally, membranes visualized on X-ray films. Densitometry analysis of individual protein bands performed using Quantitative One Image software.

### Real-time PCR analysis

Total RNA extracted from Drosophila third instar larvae with TRIzol reagent (Invitrogen). cDNA synthesized using cDNA synthesis kit (Promega) according to manufacturer’s instruction. Real-time PCR was performed with double-stranded DNA dye SYBR Green (Roche) to quantify amount of gene expression. All samples analyzed in triplicate, and mRNA levels normalized to control *Ef1α100E* values (*Elongation Factor1 alpha100*, *CG*1*873*) as previously described. *Ef1α100E* primer pair used accordingly. Information for other primer pairs is shown in (Table S7).

### Hemocyte collection and imaging

Circulating hemocytes obtained from *Drosophila* third instar larvae. Hemolymphocytes transferred into 8 µl of 1x PBS solution on adhesion microscope slides (CITOGLAS, China). Stained using the Giemsa staining kit (Bio time Institute of Biotechnology) then imaged with an Eclipse 80i Microscope (Nikon).

### Transmission Electron Microscopy (TEM)

Lymph glands of third instar larvae fixed in 2% paraformaldehyde, 2.5% glutaraldehyde, 5 mM CaCl_2_, and 0.1 mM sodium cacodylate for 24 h at 4 °C. Followed by 2 h of post-fixation in 2.5% glutaraldehyde, 0.8% osmium tetroxide, and 0.1 mM sodium cacodylate at 4 °C. Ultrathin sections (1 mm × 1 mm × 1 mm) on plastic were examined with a HITACHI (HT7700) microscope at 100 kV.

### Scavengers’ treatment

Treatment of *heix* mutant larvae with antioxidants (*N-Acetyl – L–cysteine*) (NAC)Calbiochem, USA and (*2(2,2,6,6-tetramethylpiperidin-1-oxyl-4-ylamino)-2-oxoethyl) triphenylphosphonium chloride* (Mito-TEMPO) (MT) sigma, USA scavengers in three treatments group design, one with 50 mM NAC, the other with 50 mM MT and the third with both of them (each 50 mM). Both control media (*Canton S* and *heix* mutant) contain 50 mM ethanol (EtOH). Treatment dose of VK2 is 50 mM.

### Statistical analysis

All experiments performed at least four times. Data analyzed using GraphPad Prism software. Confocal microscopy images analyzed using ImageJ and Corel draw software. Results expressed as mean ± SD, and differences determined using Analysis of variance (ANOVA). *P* values of *P < 0.05, **P < 0.01, and ***P < 0.001 were considered significant.

## Electronic supplementary material

Supplementary information
