# Supplementary material for: Vitamin K2 Prevents Lymphoma in Drosophila
Source: Sci Rep. 2017 Dec 6;7:17047. doi: 10.1038/s41598-017-17270-9 (PMC5719063; doi:10.1038/s41598-017-17270-9)
Supplement: Supplementary file 1 — Supplementary information [file 41598_2017_17270_MOESM1_ESM.pdf]

Supplementary Materials for

Vitamin K_2 Prevents Lymphoma in *Drosophila*

Maytham. A. Dragh[1,2], Zhiliang. Xu[1], Zainab. S. Al-Allak[2], and Ling Hong[1*]

1-Department of Genetics and Developmental Biology, College of Life Science and Technology, Huazhong University of Science and Technology, Wuhan, Hubei, P. R. China, 430074.

2-Department of Biology College of Life Science Misan University /Iraq.

Corresponding author

Telephone: 86-27-87792510; Fax: 86-27-87792170

Email address: lhong@mail.hust.edu.cn (Ling Hong)

This file includes:

- **Procedures:**
- **Measurement of movement speed**
- **video projector 4**
- **Figs. S1 to S9**
- **Figs. S10, Table 1**

Procedures:

Media preparation and vitamin K2 addition:

1- In rearing tubes (10 cm) long and 2 cm diameter, put 5ml of yeast/ molasses food to occupy 1 cm from the base of rearing tubes.

2- Let to cool for 12-24 h.

3- Vitamin K2 (which was previously dissolved in “ethanol alcohol in water bath 25 °C with shaking).

4- Vitamin K2 was poured in middle of media (food) to diffuse, and then let to for 10-15 min, then multiple puncturing of the media was made using needle for better penetration of the media by the vitamin.

5- Let for 24 h for complete evaporation of ethanol.

Measurement of movement speed rate and locomotion:

1- We fix 10 cm ruler on the wall of rearing tubes, which they contain, media 1 cm from the base and cotton cover 2 cm from the top.

2- Speed of the flies were measured by crossing the distance from the media to the cotton cover 7 cm (note: *heix* mutant flies were characterized by curled wings, thus unable to fly properly but jump and crew when stimulated by gentle knock on the tubes)

3- Each tube contained 5 flies; we repeat the test 10 times then calculate the mean.

4- Speed measured by sport watch. And speed rate was calculated by the following rule formula: speed rate = distance “cm”/time “sec”.

Measurement of locomotion:

1- We fix 1 cm ruler on the surface of the media

2- We put 10-20 larvae and measure locomotion by sport watch

3- Repeat the test 10 times and calculate the mean

4- Speed rate was calculated by the following rule formula:

Speed rate = distance “mm or Cm”/time “sec”.

Video projector

1- Video of mutant parents in and without VK2

2- Video of *heix* mutant Larvae.

3- Video of *heix* mutant Larvae in VK2

4-Video of (*Canton S*) Larvae W^{1118}

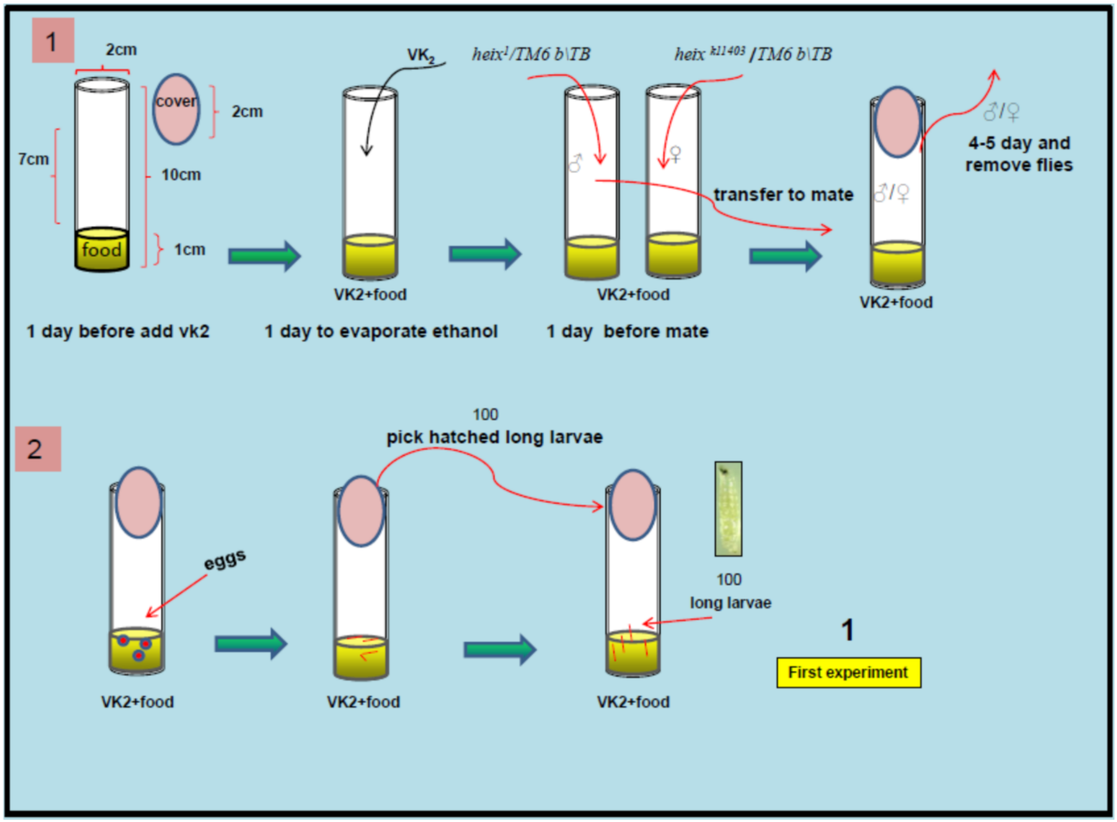

Figure S1: Preparation of vitamin K2 feeding media. Schematic diagram illustrating steps of media preparation supplemented with vitamin K2. In rearing tubes (10 cm) long and 2 cm diameter, put 5ml of yeast/ molasses food to occupy 1 cm height. Let to cool for 12-24 h. Vitamin K2 (which was previously dissolved in "ethanol alcohol in water bath 25 °C). Vitamin K2 poured in middle of media (food) to diffuse, let to for 10-15 min, then multiple puncturing media made using needle for better penetration of media by vitamin. Let 24 h for complete evaporation of ethanol. Addition of vitamin occurs next day of media preparation and introducing flies were in the next day and kept both sexes separated for 24 h then let to mate for 4-5 days. After that adult flies removed, only long larvae selected for experiment.

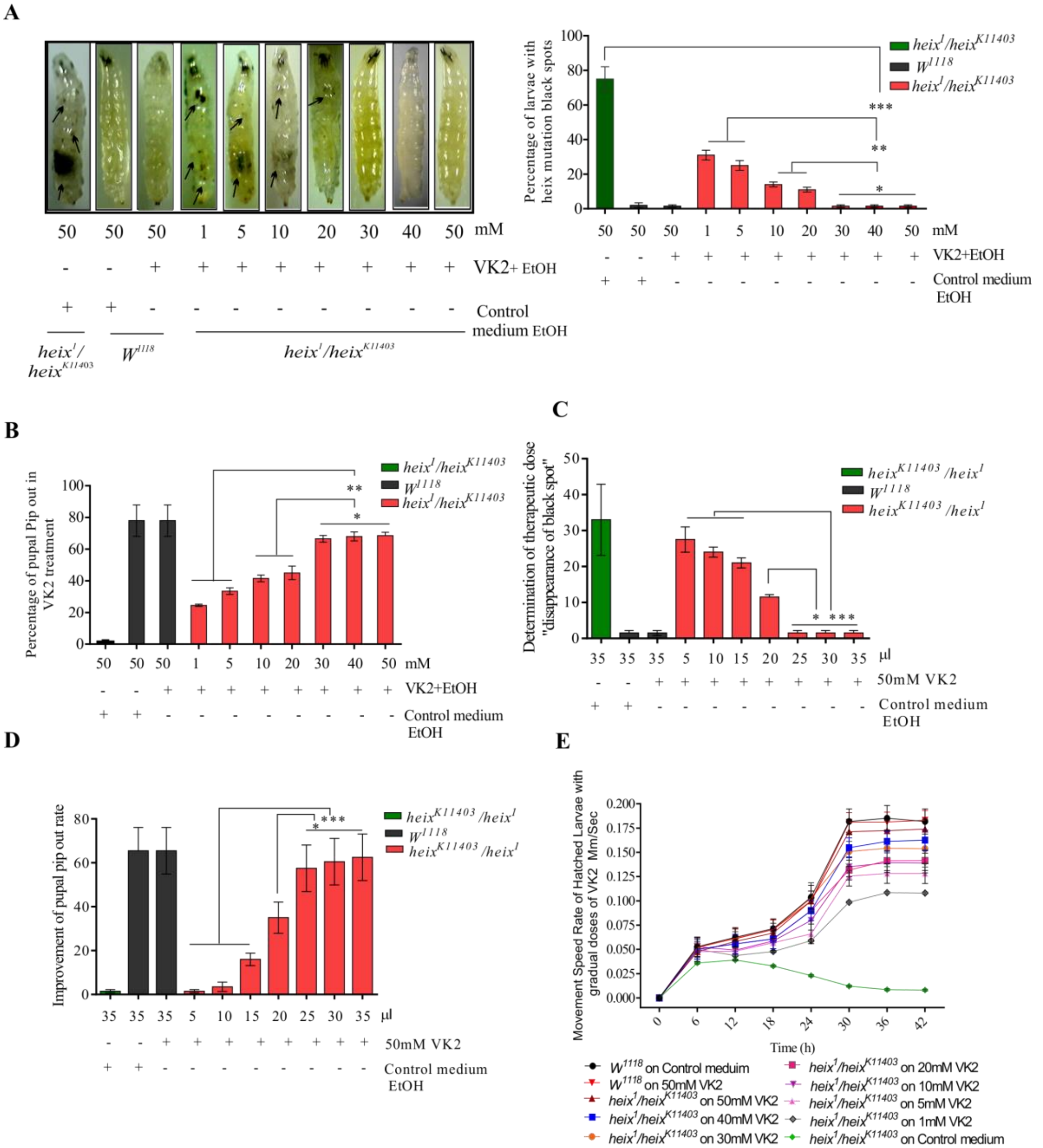

Figure S2: Determination of therapeutic dose of vitamin K2. Several gradual doses of vitamin K2 tested on *heix* mutant larvae ranged between 1mM to 50mM. (**A**) Disappearance of black spots indicator for response to treatment, concentration from 30-50mM considered significant. (**B**) Improvement of pupal pip out rate was dose dependent. (**C**) Determination of dose volume, which appear significant with 25-35µl of

50mM of VK2 associated with disappearance of black spots. (**D**) Determination of dose volume, appeared significant with 25-35µl 50mM of VK2 associated with improvement in pupal pip out rate. (**E**) Improvement of movement speed rate in hatched larvae was dose dependent, better response between 30-50mM. Control media contain 50mM Ethanol (EtOH) and treatment media contain 50mM VK2. Means normalized to control (*Canton S*). Error bars indicate SEM. Analysis of variance (ANOVA/Dunnett: * P <0.05, ** P <0.01, *** P <0.001).

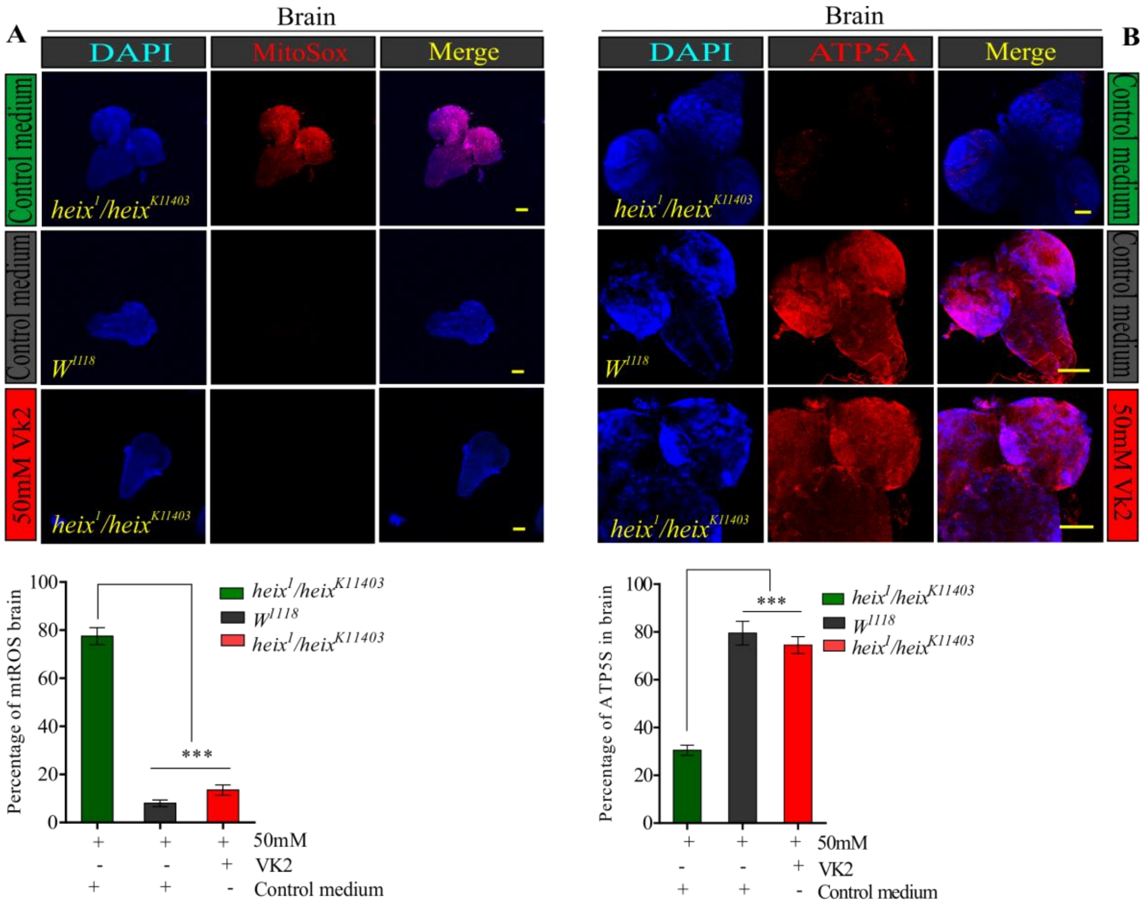

Figure S3: Vitamin K2 inhibits mitochondrial ROS "mtROS" release and improves ATP production. (**A**) Confocal microscopy showed significant brain response to treatment by lowering release of mtROS. (**B**) Confocal microscopy showed significant brain response to treatment by increase ATP production. DAPI stain (blue), mtROS staining (Mito-Sox) (red) and anti-ATP antibodies (ATP 5A). Control media contain 50mM Ethanol (EtOH) and treatment media contain 50mM VK2. Means normalized to control (*Canton S*). Error bars indicate SEM. Analysis of variance (ANOVA/Dunnett: * *P* <0.05, ** *P* <0.01, *** *P* <0.001).

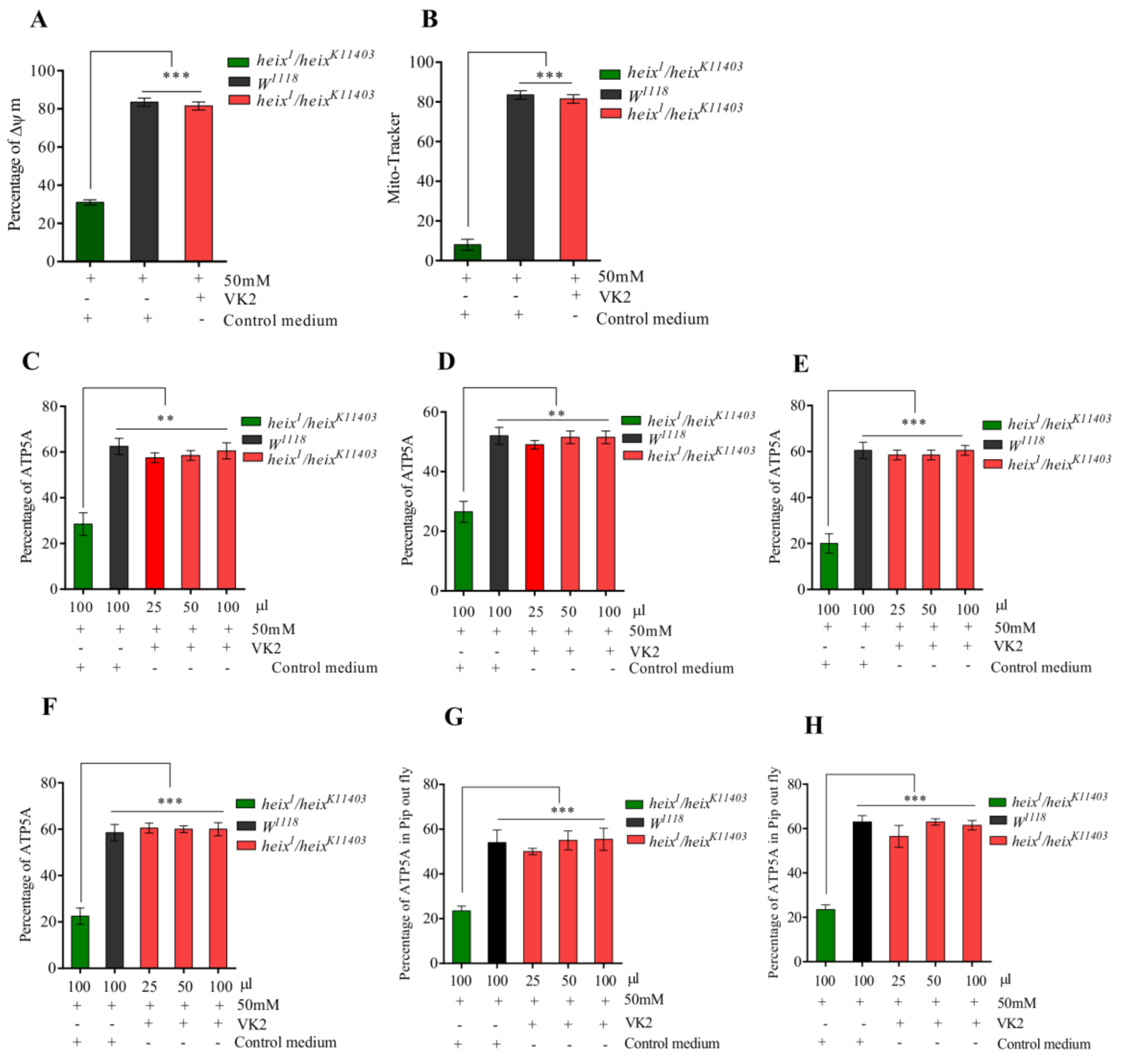

Figure S4: Significances of response to vitamin K2 treatment. (**A**) Mitochondrial membrane potential ΔΨm significant response to vitamin K2 treatment. (**B**) Mito-Tracker assay significance. (**C**, **D**, **E**, **F**) Significance of ATP production measured after 12, 24, 36, 48 h post-hatch. (**G**, **H**) Significance of ATP production measured after 24, 48 h post pip out. Control media contain 50mM Ethanol (EtOH) and treatment media contain 50mM VK2. Means normalized to control (*Canton S*). Error bars indicate SEM. Analysis of variance (ANOVA/Dunnett: * P <0.05, ** P <0.01, *** P <0.001).

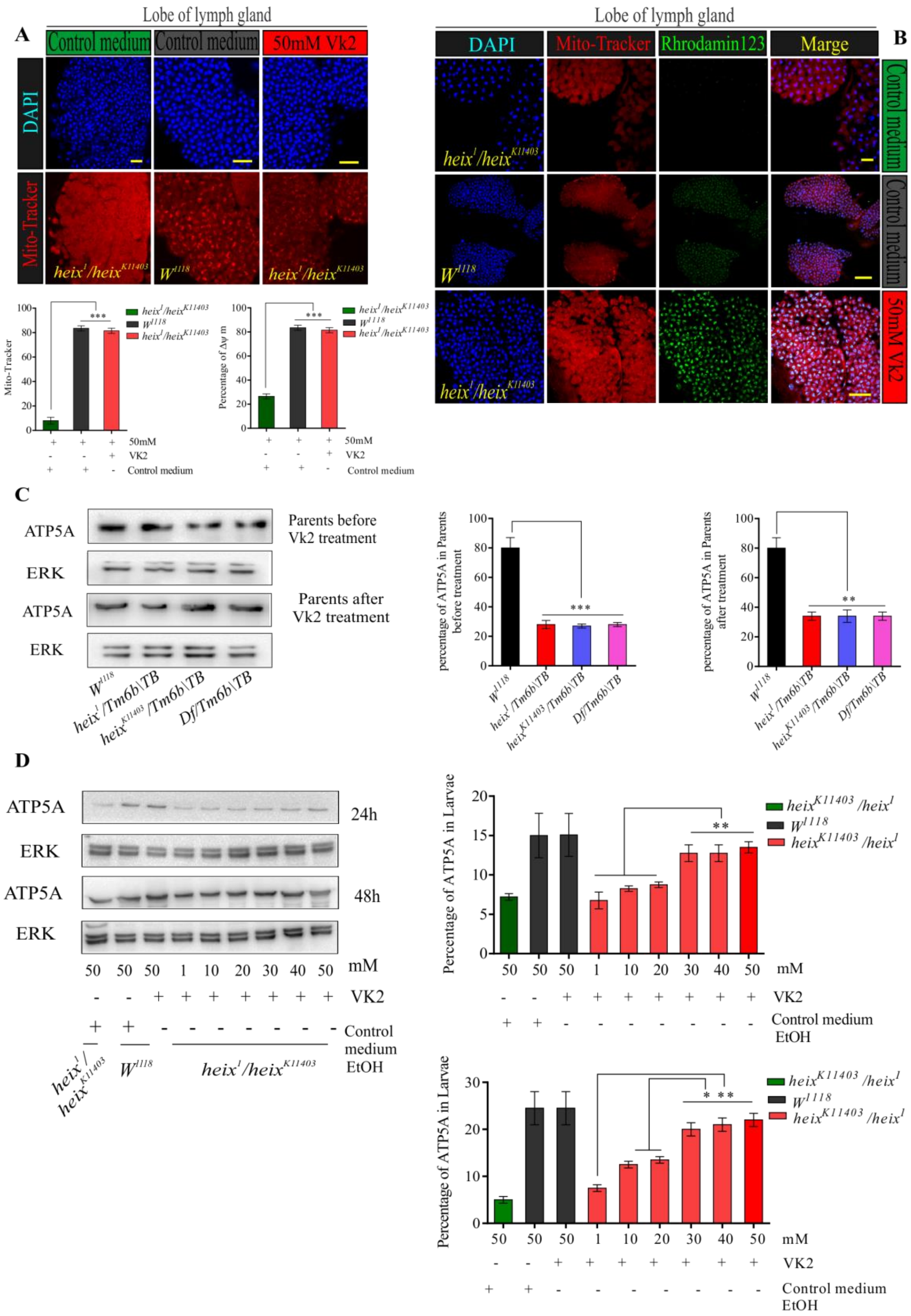
A
Lobe of lymph gland
Control medium
Control medium
50mM Vk2
DAPI
Mito-Tracker
heix1/heixK11403
W1118
heix1/heixK11403
Mito-Tracker
Percentage of Δψ m
50mM
VK2
Control medium
B
Lobe of lymph gland
DAPI
Mito-Tracker
Rhodamin123
Marge
Control medium
Control medium
50mM Vk2
C
ATP5A
ERK
ATP5A
ERK
Parents before Vk2 treatment
Parents after Vk2 treatment
W1118
heix1/Tm6b\TB
heixK11403/Tm6b\TB
Df/Tm6b\TB
percentage of ATP5A in Parents before treatment
percentage of ATP5A in Parents after treatment
D
ATP5A
ERK
ATP5A
ERK
24h
48h
mM
VK2
Control medium EtOH
heix1/heixK11403
W1118
heix1/heixK11403
Percentage of ATP5A in Larvae
heixK11403/heix1
W1118
heixK11403/heix1
Percentage of ATP5A in Larvae

Figure S5: Vitamin K2 restores mitochondrial function. (**A**) Confocal images of lymph gland labeled with Mito-Tracker indicates mitochondrial morphological defect significantly restored by VK2 treatment. (**B**) Mitochondrial membrane potential ΔΨm significantly more negative in response to treatment. (**C**) Western blot showed a significant increase in ATP production in parent flies after treatment. (**D**) Western blot analysis showed gradual increase in ATP production in 24 h and 48 h after pip out. Both measurements were significant. Control media contain 50mM Ethanol (EtOH) and treatment media contain 50mM VK2. Means normalized to control (*Canton S*). Error bars indicate SEM. Analysis of variance (ANOVA/Dunnett: * *P* <0.05, ** *P* <0.01, *** *P* <0.001).

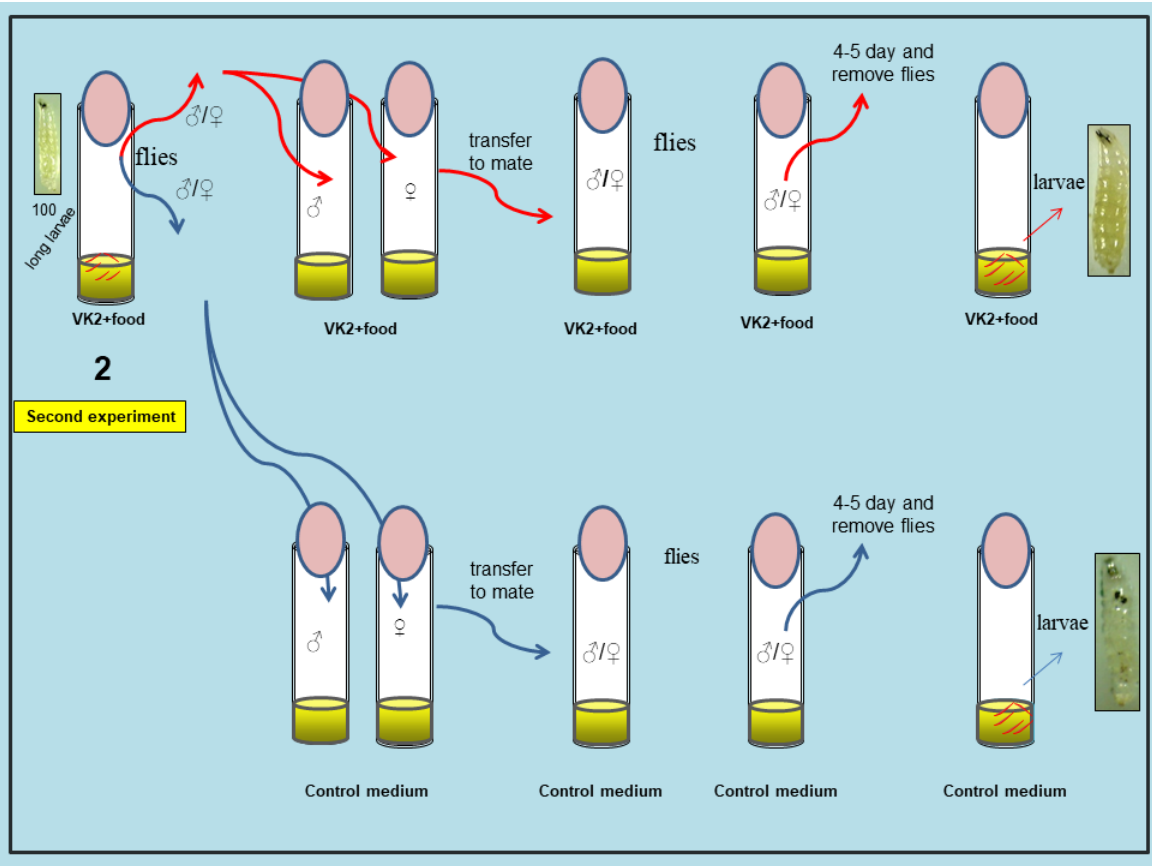

Figure S6: Second generation experiment. Schematic diagram illustrating steps of second experiment design. Beginning with selection of 100 long larvae *heix* mutant fed with VK2 complete disappearance of black spots, then Improvement of pupal pip out. Flies separated in to male and female in new tube. They divided in to two groups and let to mate, red arrows group continued feeding on VK2 and showed no reappearance of lymphoma phenotypes (no black spots and good survival signs), and blue arrows group stop feeding on VK2, showed recurrence of lymphoma phenotype (black spot and poor survival signs).

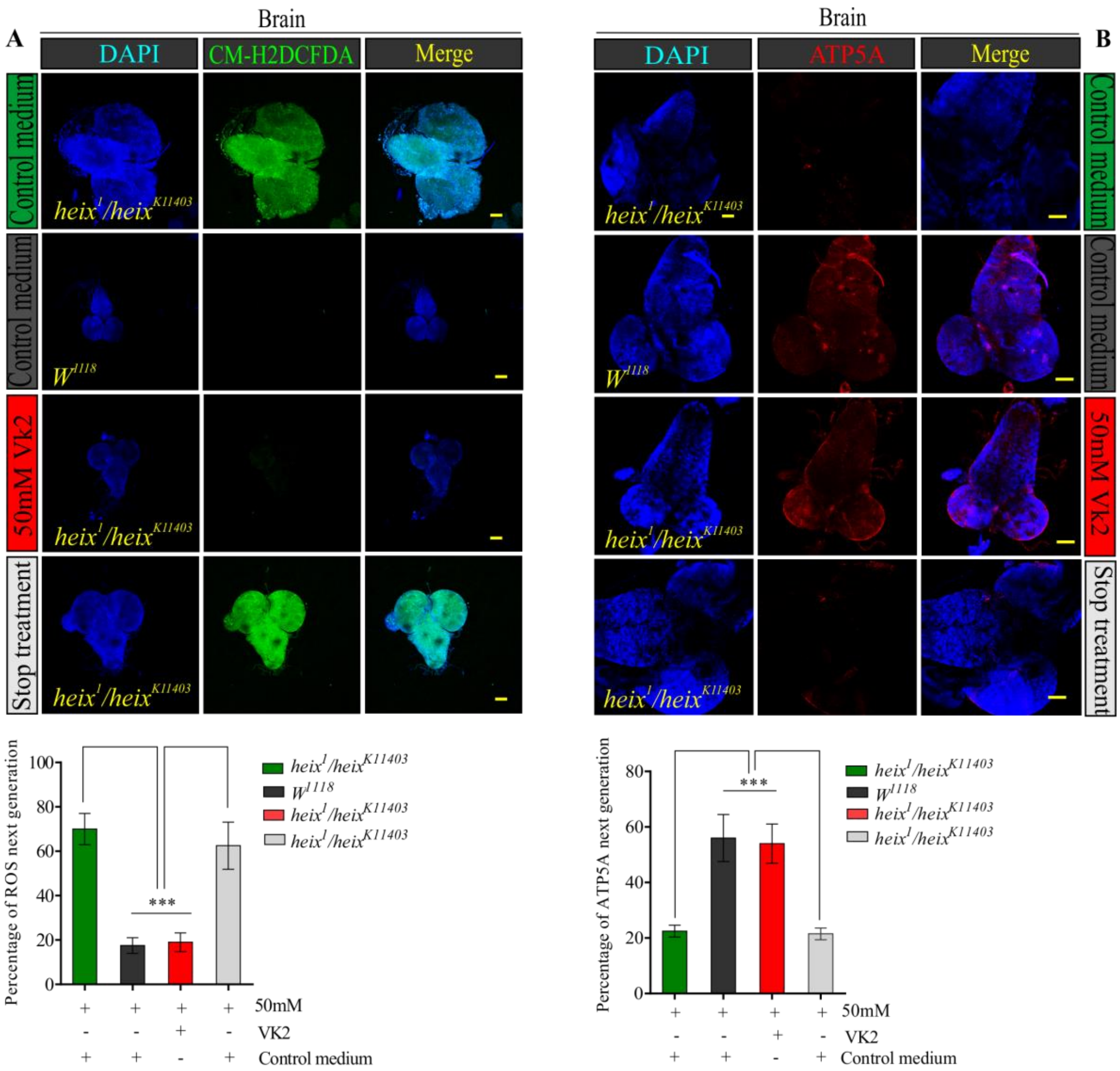

Figure S7: Prevention of lymphoma was vitamin K2 dependent. Second generation experiment. Recurrences of lymphoma occur after stop treatment with vitamin K2. (**A**) Recurrence of ROS release significant in brain. DAPI stain (blue) and ROS staining (CM-H2DCFDA) (green). (**B**) Inhibition of ATP production, which was significant. DAPI stain (blue) and anti-ATP antibodies (ATP5A). Control media contain 50mM Ethanol (EtOH) and treatment media contain 50mM VK2. Means normalized to control (*Canton S*). Error bars indicate SEM. Analysis of variance (ANOVA/Dunnett: * *P* <0.05, ** *P* <0.01, *** *P* <0.001).

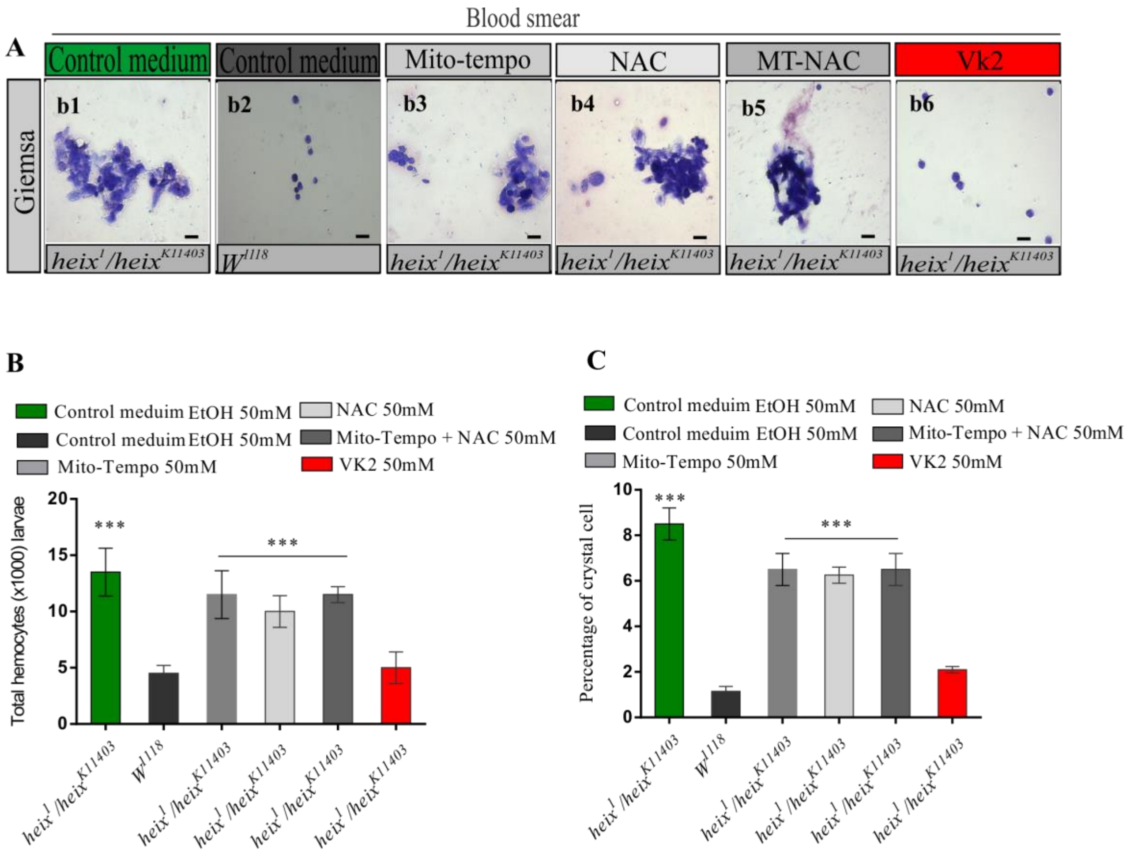

Figure S8: Response is VK2 related. Treatment of *heix* mutant larvae with antioxidants N-Acetyl-L-cysteine (NAC) as anti-ROS, and (mito-TEMPO) (MT) as anti-mtROS. (**A**) Blood smear revealed that none of scavengers used in separate or mixed

treatment inhibit hemocyte and crystal cell proliferation. *heix* mutant on control medium with hemocyte proliferation, control (*Canton S*) on control medium normal was normal, *heix* mutant on VK2 treatment, picture showed reduction in hemocyte count reflecting response to VK2. (**B**) Treatment with ROS scavengers didn't reduce total hemocyte count as compared with treatment with VK2. (**C**) Persistent elevated count of crystal cell treated with ROS scavengers. Control media of control (*Canton S*) and *heix* mutant contain 50mM Ethanol (EtOH), and treatment media contain 50mM of each scavenger's. Means normalized to control (*Canton S*). Error bars indicate SEM. Analysis of variance (ANOVA/Dunnett: * *P* <0.05, ** *P* <0.01, *** *P* <0.001).

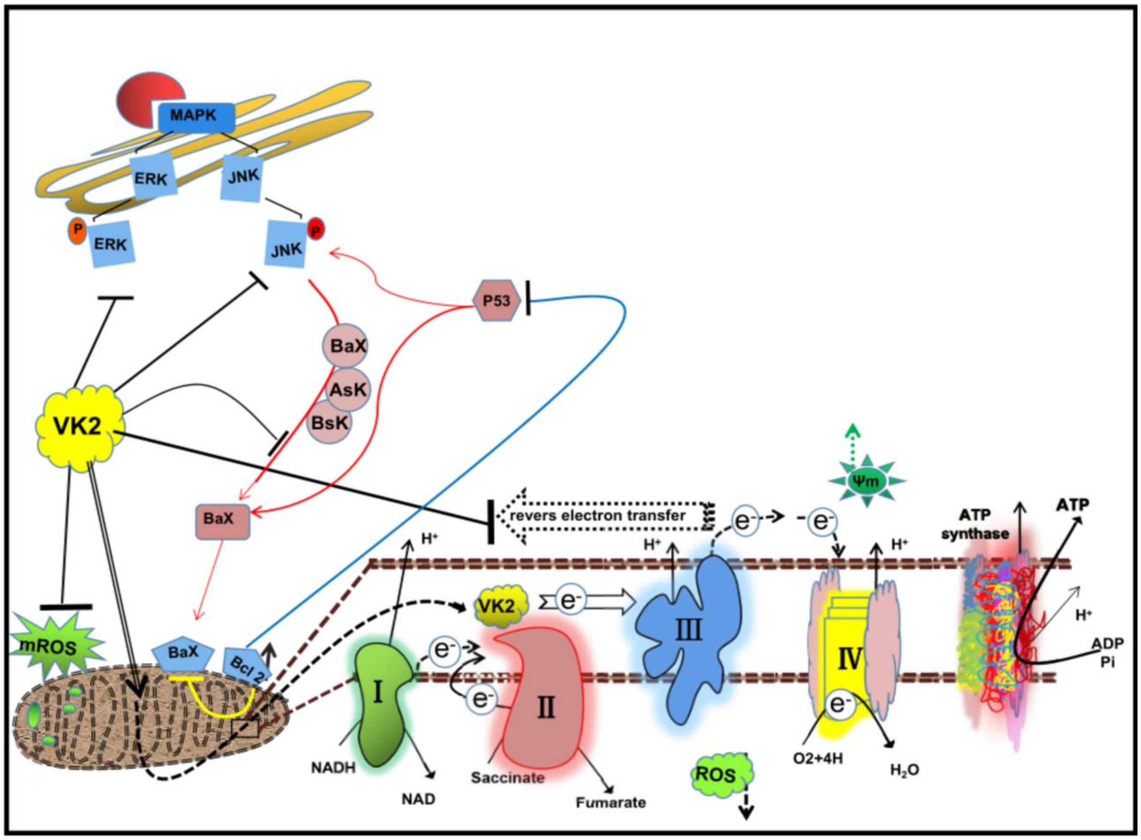

Figure S9: "Effect of vitamin K2". A schematic diagram illustrating the effects of vitamin K2, it possesses the ability to inhibit and stop activation of JNK and ERK

pathways as well as reduce release of mtROS from complex I and Ш of ETC in mitochondrial membrane through acting as an electron transport carrier. Preventing electron reverses and increase mitochondrial membrane potential lead to increase ATP production.

SYMBOL	ANNOTATION SYMBOL	FORWARD PRIMER	REVERSE PRIMER	INTRON (BP)	AMPLICON SIZE (BP)
Upd1	CG5890	TAATGCGTAGTTGGTTGGC	AGCAGGACGAGTAGGATGG	6124	77
Upd2	CG6075	CTTCGTGGGCAGCTTCTCAT	GCTGATCCTTGCGGAACTTG	5967	101
Upd3	CG5439	ATGTCCCAGTTTGCCCTCT	TCCTTTGGCGTTTCTTGC	5706	276
HoP	CG1594	GATTTCCAGCCGCACAGAGG	GCCAAACGACGTTCCACCAG	270	189
Mkp3	CG14080	TGGTGATGGTGGCTGGAGCT	ACGATAATCAGACGCACAGCAAAG	92	163
SPE	CG16705	CAGGGTTGAGGGCTTGGTGAA	CCGCCGCAGTTGAAAGTGTAGG	216	261
SPZ	CG6134	ATGGCGGTTCGTTTCGG	CCAATGATGAGGGAGTTGTCC	237	140
Myd88	CG2078	AACGAGCCAACTTGTCCCAGAA	CTGATCGTCGTGCTCACAGAGG	73	217
cact	CG5848	TATCAATGTGACCCGCCTCG	CCACGGAAATCCATGAGGC	8101	159
DIf	CG6794	CACAGGAGACCACGATGATGAC	GGAGGAGCCGACAAGCAAT	0	161
DI	CG6667	AGATGGGCTCCGAAACTACCG	GCTGCAAGAAGGGCGTCTGTA	69	282
Drs	CG10810	AATCATTTACCAAGCTCCGTGAG	CAGCGAAGAGGGCGAACA	0	73
IMD	CG5576	TCCCGCATCACCTGCTTCC	GCACTTCGGCTCCGTCTACAAC	0	237
Dome	CG7486	CAGAGCAGCATATCCATTTACCG	CGTCTACGCATCCAACAGCAT	68	235
BsK	CG5680	AGTTTCGGACGGTTGGC	CTACCTTTATGATGACTCCCTATGT	61	243
Rel	CG11992	CAAACTGCACCGGATGACG	GAAGCGGACGCCCAAAA	0	76
Dpt	CG12763	CCGTCGCCTTACTTTGCTG	CCGCCTCCCTGAAGATTGA	0	115
BaX	CG5633	TCTCCTTGTCCGCCCTTCT	ACCAGCATCCCACCCAGATA	210	150
BcL-2	CG5146	GGTTGACGCTGTTCGTGACTATC	TGCGCCGACACACGTTT	175	123
P53	CG5467	TCAGCAGTTCGGGTCTCC	GTTCGTGTTGTTGCCTCC	0	175
AsK	CG5478	CGAAATCCGTCCAAGAAGAGC	CGCCGTAGTGATAGGAAAGC	201	167

Figure S10: Table 1. Information of primers used in this study.
